# The Glycogen Synthase Kinase 3α and β Isoforms Differentially Regulates Interleukin-12p40 Expression in Endothelial Cells Stimulated with Peptidoglycan from *Staphylococcus aureus*

**DOI:** 10.1371/journal.pone.0132867

**Published:** 2015-07-22

**Authors:** Ricarda Cortés-Vieyra, Octavio Silva-García, Javier Oviedo-Boyso, Alejandro Huante-Mendoza, Alejandro Bravo-Patiño, Juan J. Valdez-Alarcón, B. Brett Finlay, Víctor M. Baizabal-Aguirre

**Affiliations:** 1 Molecular Immunology and Signal Transduction Laboratory, Centro Multidisciplinario de Estudios en Biotecnología, Facultad de Medicina Veterinaria y Zootecnia, Universidad Michoacana de San Nicolás de Hidalgo, Carretera Morelia-Zinapécuaro, La Palma, Tarímbaro, Morelia, Michoacán, México; 2 Michael Smith Laboratories, The University of British Columbia, Vancouver, British Columbia, Canada; Institute of Biochemistry and Biotechnology, TAIWAN

## Abstract

Glycogen synthase kinase 3 (GSK3) is a constitutively active regulatory enzyme that is important in cancer, diabetes, and cardiovascular, neurodegenerative, and psychiatric diseases. While GSK3α is usually important in neurodegenerative and psychiatric diseases GSK3β is fundamental in the inflammatory response caused by bacterial components. Peptidoglycan (PGN), one of the most abundant cell-wall structures of Gram-positive bacteria, is an important inducer of inflammation. To evaluate whether inhibition of GSK3α and GSK3β activity in bovine endothelial cells (BEC) regulates the expression of the pro-inflammatory cytokine IL-12p40, we treated BEC with SDS-purified PGN from *Staphylococcus aureus*. We found that PGN triggered a TLR2/PI3K/Akt-dependent phosphorylation of GSK3α at Ser21, GSK3β at Ser9, and NF-κB p65 subunit (p65) at Ser536, and the phosphorylation of GSK3α was consistently higher than that of GSK3β. The expression of IL-12p40 was inhibited in BEC stimulated with PGN and pre-treated with a specific neutralizing anti-TLR2 antibody that targets the extracellular domain of TLR2 or by the addition of Akt-i IV (an Akt inhibitor). Inhibition of GSK3α and GSK3β with LiCl or SB216763 induced an increase in IL-12p40 mRNA and protein. The effect of each isoform on IL-12p40 expression was evaluated by siRNA-gene expression silencing of GSK3α and GSK3β. GSK3α gene silencing resulted in a marked increase in IL-12p40 mRNA and protein while GSK3β gene silencing had the opposite effect on IL-12p40 expression. These results indicate that the TLR2/PI3K/Akt-dependent inhibition of GSK3α activity also plays an important role in the inflammatory response caused by stimulation of BEC with PGN from *S*. *aureus*.

## Introduction

*Staphylococcus aureus* causes important infectious diseases in animals and humans because it expresses a wide arrange of virulence factors and cell-wall associated structures that are responsible for damaging tissues [1]. One of the main cell wall structures of *S*. *aureus* is the peptidoglycan (PGN) that activates the innate immune system of the host and promotes inflammation [2] via the PI3K/Akt signaling pathway [3–5]. While it has been proposed that the intracellular receptors NOD1/2, but not TLR2, are the main proteins involved in PGN signaling [2], some studies have shown that TLR2 is a major receptor that senses PGN. For example, treatment of RAW264.7 macrophages with PGN induced the TLR2-dependent recruitment of p85α, Rac1 and Ras that mediates IKKα/β-NF-κB activation, and COX2 expression through the Rac1/PI3K/Akt and Ras/Raf1/Erk1-2 signaling pathways [3, 6]. Also, in BV-2 microglia, PGN activates the TLR2/MyD88/PI3K/Akt pathway, which leads to IκBα degradation, phosphorylation of NF-κB p65 subunit (p65) at Ser536, and expression of pro-inflammatory cytokines, iNOS, and COX2 [4]. Moreover, PGN binds to TLR2 in fibroblasts and activates the FAK/PI3K/Akt signaling and the transcription factor AP-1, leading to an increase in IL-6 expression [5].

The phosphoinositide 3-kinase/Akt (PI3K/Akt) signaling pathway mediates a variety of cellular responses such as survival, proliferation, differentiation, apoptosis, and as mentioned above, inflammation [7, 8]. Activation of PI3K/Akt pathway leads to the PI3K-dependent synthesis of phosphatydilinositol-3,4,5-triphosphate (PIP_3_) and phosphorylation of Akt at Thr308 and Ser473 by the constitutively active PDK1 [9, 10] and mTORC2 [11, 12]. Akt in turn regulates the activity of a wide range of substrates, among which glycogen synthase kinase 3 (GSK3) is important in the modulation of the inflammatory response [8, 13]. GSK3 refers to two mammalian paralogs that are commonly called GSK3α and GSK3β isoforms [14], which are constitutively active and can be inactivated by phosphorylation at Ser21 (GSK3α) or Ser9 (GSK3β) by Akt [15]. Since its discovery, GSK3β has been shown to be involved in the regulation of many cellular functions including growth, differentiation, embryonic development, cell cycle progression, apoptosis [16, 17] and in the inflammatory response caused by bacterial infection through the regulation of NF-κB activity [13, 15]. In regard to GSK3α most of the glucose/glycogen homeostasis appears to depend mainly on this isoform, with a minor contribution of GSK3β in skeletal muscle [18–20]. Also, GSK3α plays a potential role as a regulatory enzyme of the central nervous system [21]. This isoform, but not GSK3β, has recently been identified in the maintenance and/or proliferation of Th17 cells stimulated with the pro-inflammatory cytokine IL-1 [22]. However, participation of GSK3α in the modulation of inflammation, triggered by microbial products has not been well documented. Studies in murine models indicate that both isoforms of GSK3 are not physiologically redundant [16, 18, 23]. Recently, it was found that LiCl inhibition of GSK3α in lipopolysaccharide (LPS)-activated neutrophils and in the murine dorsal air-pouch model lead to a large increase in TNF-α secretion by affecting the translational mechanism of the TNF-α protein without altering its mRNA levels [24]. Furthermore, in a previous report we demonstrated that internalization of *S*. *aureus* by endothelial cells is associated with the PI3K/Akt activity [25]. Although the correlation between *S*. *aureus* internalization and GSK3α or GSK3β activity was not analyzed in that report, we indeed observed a higher phosphorylation of GSK3α at Ser21 than GSK3β at Ser9 [25]. Thus, it is likely that GSK3α has also regulatory functions in the inflammatory response induced by *S*. *aureus*.

Interleukin (IL)-12 is an important pro-inflammatory cytokine because its expression during bacterial infection regulates the innate response and determines the type and duration of the adaptive immune response [26, 27]. Structurally, this cytokine is a heterodimer composed of two subunits designated p35 and p40 linked by disulfide bonds [28, 29]. The *IL-12p40* gene is highly inducible by microbial products such as LPS, lipoteichoic acid (LTA) and PGN via Toll-like receptor signaling and NF-κB activation [27]. Antigen-presenting cells and phagocytic cells are the primary producers of IL-12 [27], although human endothelial cells also produce it [30]. Despite IL-12 is essential for host defense, its overexpression can cause persistent inflammation giving rise to autoimmune disorders. To counterbalance the action of IL-12, immune cells produce IL-10 that decreases NF-κB and AP-1 activity, and at the same time increases CREB activity [31, 32].

Stimulation of human monocytes and peripheral blood mononuclear cells (PBMCs) with agonists of TLR2 (LTA from *Streptococcus pneumoniae*), TLR4 (LPS or synthetic lipid A), TLR5 (flagellin from *Salmonella* Typhimurium), or TLR9 (human CpG), reduced the expression of IL-12p40 through the inhibition of GSK3β with no participation of the GSK3α isoform [32]. In contrast, data presented in this work indicate that stimulation of bovine endothelial cells (BEC) with PGN from *S*. *aureus* modulates the expression of IL-12p40 through the inhibition of GSK3α and GSK3β. Interestingly, inhibition of GSK3α with pharmacological drugs or its gene expression silencing with interference RNA in BEC stimulated with PGN produced a marked increase in the expression of IL-12p40. In similar experiments, directed to GSK3β, we observed a reduced expression of IL-12p40. In both cases the mechanism involved the activation of the TLR2/PI3K/Akt signaling pathway. Altogether, the biochemical evidence presented indicates that both isoforms of GSK3 differentially modulate the expression of IL-12p40, a pro-inflammatory cytokine.

## Materials and Methods

### Media and Chemicals

F-12 Ham (HF-12) of Dulbecco’s modified Eagle’s medium, bovine serum albumin (BSA), trypsin-EDTA, Igepal CA-930, PGN from *S*. *aureus* 77140, Wortmannin (Wort), Akt-i IV, LY294002 (LY), SB216763 (SB), NaCl, LiCl, puromycin, and Bradford reagents were purchased from Sigma-Aldrich, Inc. (St. Louis, MO, USA). Fetal calf serum (FCS) was acquired from Equitech-Bio, Inc. (Kerrville, TX, USA). A cocktail of sodium penicillin G, streptomycin sulfate, and amphotericin B was purchased from Gibco-BRL (Gaithesburg, MD, USA). Akt Inhibitor II, D-3-Deoxy-2-O-methyl-myo-inositol 1-[(R)-2-methoxy-3-(octadecyloxy) propyl hydrogen phosphate (SH-5) was acquired from Calbiochem (Darmstadt, Germany). Halt Phosphatase inhibitor cocktail was purchased from Thermo Fisher Scientific (Waltham, Massachusetts, USA). Protease inhibitor cocktail was acquired from GE Healthcare Bio-sciences (Little Chalfont, UK). Trizol reagent and EXPRESS One-Step SYBR GreenER Universal Kit were purchased from Invitrogen (Carlsbad, CA, USA). Bovine Interleukin 12 (IL-12/p40) TSZ ELISA kit was purchased from Biotang (Massachusetts, USA). All other reagents were acquired from Sigma-Aldrich.

### Antibodies

Rabbit polyclonal antibodies against the extracellular domain of TLR2 (N-17; sc-8689), the isotype unspecific IgG, and goat anti-rabbit IgG-HRP were purchased from Santa Cruz Biotechnology, Inc. (Santa Cruz, CA, USA). Rabbit polyclonal antibodies against phospho-glycogen synthase (Ser641) and rabbit monoclonal antibodies against phospho-Akt (Ser473), phospho-GSK3α (Ser21), phospho-GSK3β (Ser9), phospho-p65 (Ser536), Akt, GSK3β, and NF-κB p65 subunit were purchased from Cell Signaling Technology (Boston, MA, USA).

### Cell Line and Culture Conditions

The endothelial cell line used as a model cell in this study was obtained from bovine umbilical veins and immortalized by transfection with an expression vector containing the E6-E7 oncogenes of human papillomavirus 16 (BVE-E6E7) [33]. This immortalized bovine endothelial cell line, called BEC in this study, was grown and maintained in HF-12 supplemented with 10% FCS and a cocktail of sodium penicillin G, streptomycin sulfate, and amphotericin B, unless otherwise noted.

### Purification of PGN

We eliminated lipopeptides from commercial PGN preparations from *S*. *aureus* (Sigma-Aldrich) as previously described by Dziarski and Gupta (2005) [34]. Briefly, 2 mg of PGN per mL were treated with 8% sodium dodecyl sulfate (SDS) at 90°C for 30 min, followed by 10 washes with H_2_O to remove SDS. The concentration of purified PGN was calculated spectrophotometrically at 450 nm by adjusting the value of the dispersion to the same value obtained from commercial PGN that was prepared at 1 μg/mL.

### BEC Transfection

BEC (2 x 10^5^) were grown in six-well culture plates with HF-12K without serum and antibiotics for 24 h. Then, each well was transfected with the X-tremeGENE HP DNA Transfection Reagent kit (Roche). We used either 3 μg of siRNA-GSK3α plasmids (Sigma Aldrich, clone IDs, pLKO.1-GSK3α1: NM_019884.1-948s1c1; pLKO.1-GSK3α2: NM_019884.1-567s1c1, and pLKO.1-GSK3α3: NM_019884.2-1207s1c1) or siRNA-GSK3β plasmids (pLKO.1-GSK3β1 and pLKO.1-GSK3β2) that were gifts from Alex Toker (Addgene plasmid # 32496 and # 32497). Control cells were transfected with 3 μg of the pLKO.1 plasmid (pLKO.1) that was a kind gift from Bob Weinberg (Addgene plasmid # 8453). For GSK3α the targeting sequences were: 5’-TACATCTGTTCTCGCTACTA-3’ (nucleotides 1535–1554, pLKO.1-GSK3α1); 5’-CCAGGACAAGAGGTTCAAGAA-3’ (nucleotides 1153–1173, pLKO.1-GSK3α2); 5’-CCTGGACAAAGGTGTTCAAAT-3’ (nucleotides 1788–1808, pLKO.1-GSK3α3); 5’-GAAGTCAGCTATACAGACACT-3’ (nucleotides 587–607, pLKO.1-GSK3β1) and 5’-GAAAGCTAGATCACTGTAACA-3’ (nucleotides 735–755, pLKO.1-GSK3β2). After 24 h, the culture medium was changed to HF-12K with serum plus 0.8 μg/mL of puromycin and cells were incubated for 15 days to select for stable transfections. BEC were recovered and sub-cultured in six-well culture plates in HF-12K without serum, incubated for 24 h and stimulated with PGN, as described below.

### Protein Extraction and Western Blot Assays

To test for the relative abundance of phosphorylated and non-phosphorylated proteins, BEC were grown in six-well tissue culture plates (Ultra Cruz) to approximately 90% confluence before serum starvation for at least 4 h. Total protein (cytosolic plus nuclear) from control and treated cells was obtained by washing the cells 2X with cold PBS and lysing them with 100 μl of a cold lysis buffer containing 20 mM Tris-HCl, pH 7.5, 150 mM NaCl, 1% Igepal CA-930, 10 mM Na-pyrophosphate, 50 mM NaF and 1 mM Na-orthovanadate supplemented with 1X protease inhibitor cocktail and 1X phosphatase inhibitor cocktail, which were added immediately before lysing the endothelial cells. The lysates were centrifuged at 16,000 xg for 20 min at 4°C and the supernatant was transferred to ice-cold Eppendorf tubes. Protein concentration was measured by the Bradford method [35] using BSA as standard. Then, 30–40 μg of protein was separated by electrophoresis in 10% sodium dodecyl sulfate-polyacrylamide gels and electroblotted in a wet chamber onto 0.45 μm nitrocellulose membrane (Bio-Rad) at 250–300 mA for 1 h. Membranes were probed with primary polyclonal antibodies to phosphorylated forms of Akt, GSK3α, GSK3β, or p65. Then, membranes were stripped, reprobed with secondary monoclonal antibodies to the non-phosphorylated form of Akt or polyclonal antibodies to GSK3β or p65 as controls of protein loading, and detected with the Immobilon Western Chemiluminescent HRP substrate kit from Millipore (Billerica, MA, USA).

### RNA Extraction and qRT-PCR

To analyze the relative expression of IL-12p40 mRNA, BEC were grown in six-well culture plates to approximately 90% confluence before serum starvation for at least 4 h. Then 10 μg/mL of PGN was added to the cultured cells, centrifuged at 130 xg for 5 min and incubated for 2, 4 or 8 h at 37°C in 5% CO_2_, or pretreated with 10 μM SH-5, 10 μM SB216763, 10 mM NaCl, 10 mM LiCl or 5 μg/mL anti-TLR2 for 1 h, or with Akt-i IV for 0.5 h followed by washing with HF-12 without serum and stimulation with 10 μg/mL PGN, centrifuged at 130 xg for 5 min and incubated for 4 h at 37°C in 5% CO_2_. At the end of the incubation period, BEC were washed 2X with cold PBS and total RNA was extracted using 1 mL Trizol of reagent following the isolation procedure described by the supplier. One-step reverse transcription and real-time quantitative PCR (qRT-PCR) was performed using the EXPRESS One-Step SYBR GreenER Universal Kit and the real-time StepOnePlus thermocycler from Applied Biosystems. Each reaction was performed with 100 ng/μL of RNA under the standard 20 μL reaction provided by Invitrogen. The one-step cycling program conditions was: 58°C (for *IL12p40*) and 50°C (for *β-actin*) for 5 min (cDNA synthesis); 95°C for 2 min; 40 cycles at 95°C for 15 s and 55°C (for *IL12p40*), 60°C (for *β-actin*) for 1 min. The oligonucleotide primers used were based on the sequences published by Konnai et al. 2003 [36]. Amplification of the expected single products (186 pb for *IL12p40* and 227 pb for *β-actin*) was confirmed by visualization on 1% agarose gels stained with ethidium bromide. Relative transcript levels of IL-12p40 mRNA were calculated with the delta-delta C_t_ method, using β-actin as the reference gene.

### Measurement of IL-12p40 protein levels

Bovine IL-12p40 protein in culture supernatants was measured by sandwich ELISA, according to manufacturer instructions (Biotang).

### Statistical analysis

The relative abundance of phosphorylated proteins was quantitated by densitometric analysis with the Image Processing and Analysis in Java Program ImageJ (http://rsbweb.nih.gov/ij). To calculate the densitometric values, the intensity of the phosphorylated band was divided by the intensity of the non-phosphorylated ones. These intensity values were referred to a value of 1.0 that was arbitrarily assigned to the untreated control. The statistical significance of triplicate blots, IL-12p40 mRNA levels and IL-12p40 protein levels were evaluated with the Tukey multiple-comparison test and One-Way analysis of variance (ANOVA) by using the SIGMASTAT program version 3.0 (SPSS Inc., Chicago, IL, USA). P values <0.05 or <0.01 or <0.001 were considered statistically significant.

## Results

### Phosphorylation of Akt, GSK3α and GSK3β in BEC Stimulated with PGN Depends on TLR2

**Fig 1:**
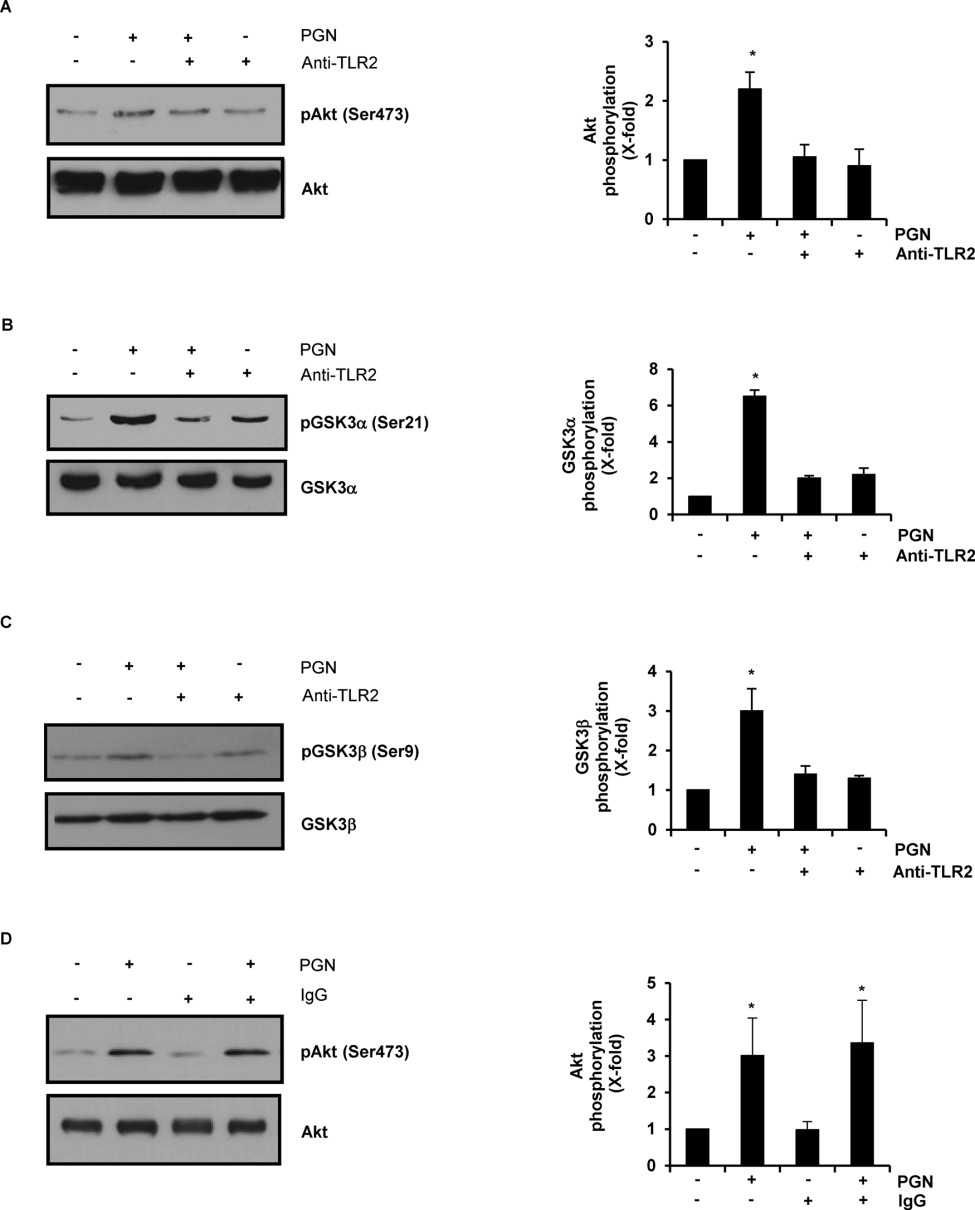
Peptidoglycan (PGN) induces phosphorylation of Akt, GSK3α and GSK3β in bovine endothelial cells (BEC) through TLR2 activation. A, B, C) BEC were either stimulated with 10 μg/mL of PGN for 30 min, pre-incubated with 5 μg/mL of neutralizing antibody against the extracellular domain of TLR2 (anti-TLR2) for 60 min and stimulated with 10 μg/mL of PGN for 30 min or pre-incubated with 5 μg/mL of anti-TLR2 for 60 min. D) BEC were either stimulated with 10 μg/mL of PGN for 30 min, pre-incubated with 5 μg/mL of an isotype anti-IgG for 60 min or pre-incubated with 5 μg/mL of anti-IgG for 60 min and stimulated with 10 μg/mL of PGN for 30 min. As control in all cases A-D, BEC were left untreated (-). After treatments, protein extracts were analyzed by western blot and probed with monoclonal antibodies against the phosphorylated forms of Akt1 (pAkt Ser473), GSK3α (pGSK3α Ser21) or GSK3β (pGSK3β Ser9). Blots were stripped and reprobed with an antibody that recognizes the nonphosphorylated forms of Akt (A, and D), GSK3α (B) or GSK3β (C) to verify equal protein loading. Blots are representative of three independent experiments. Graphs on the right panel indicate the band intensity obtained by densitometric analysis. Results are expressed as the mean ± S.E.M. (*n* = 3). *p < 0.05, compared with the untreated control.

In macrophages, microglia and fibroblasts PGN induces the expression of pro-inflammatory molecules by activation of the PI3K/Akt signaling pathway in a TLR2-dependent manner [3–6], and Akt-dependent inhibition of GSK3β by phosphorylation at Ser9 [15]. Therefore, we first decided to explore the involvement of TLR2 in the phosphorylation of Akt, GSK3α and GSK3β in BEC stimulated with PGN. We observed that in BEC stimulated with 10 μg/mL of PGN for 15 min (S1A and S1B Fig), the relative abundance of phosphorylated Akt (∼2-fold), GSK3α (∼6-fold) and GSK3β(∼3-fold) were inhibited in BEC pre-treated with a neutralizing antibody against the extracellular domain of TLR2 (Fig 1A, 1B and 1C). Phosphorylation of Akt was not affected in BEC pre-treated with an unspecific isotype IgG (Fig 1D). It has been reported that PGN induces the expression of TLR2 in microglia and fibroblasts [4, 5]. To rule out this effect, we evaluated the relative abundance of TLR2 in BEC stimulated with PGN at different times and no change was observed in the levels of TLR2 (S1C Fig). A complete loss of Akt phosphorylation was observed when BEC were pre-treated with LY-294002 (LY, an inhibitor of PI3K) and then stimulated with PGN (S1D Fig). Analysis of GSK3α/β phosphorylation confirmed that stimulation of BEC with 10 μg/mL of PGN for 15 to 60 min induced a strong increase in GSK3α phosphorylation at Ser21 compared with the minor increase in GSK3β phosphorylation at Ser9 (S2A and S2B Fig). These results indicate that 1) PGN induces TLR2-dependent phosphorylation of Akt, GSK3α and GSK3β in endothelial cells and 2) GSK3α was consistently the isoform with the highest level of phosphorylation.

### PGN Activates the PI3K/Akt-Dependent Phosphorylation of GSK3α and GSK3β in BEC

**Fig 2:**
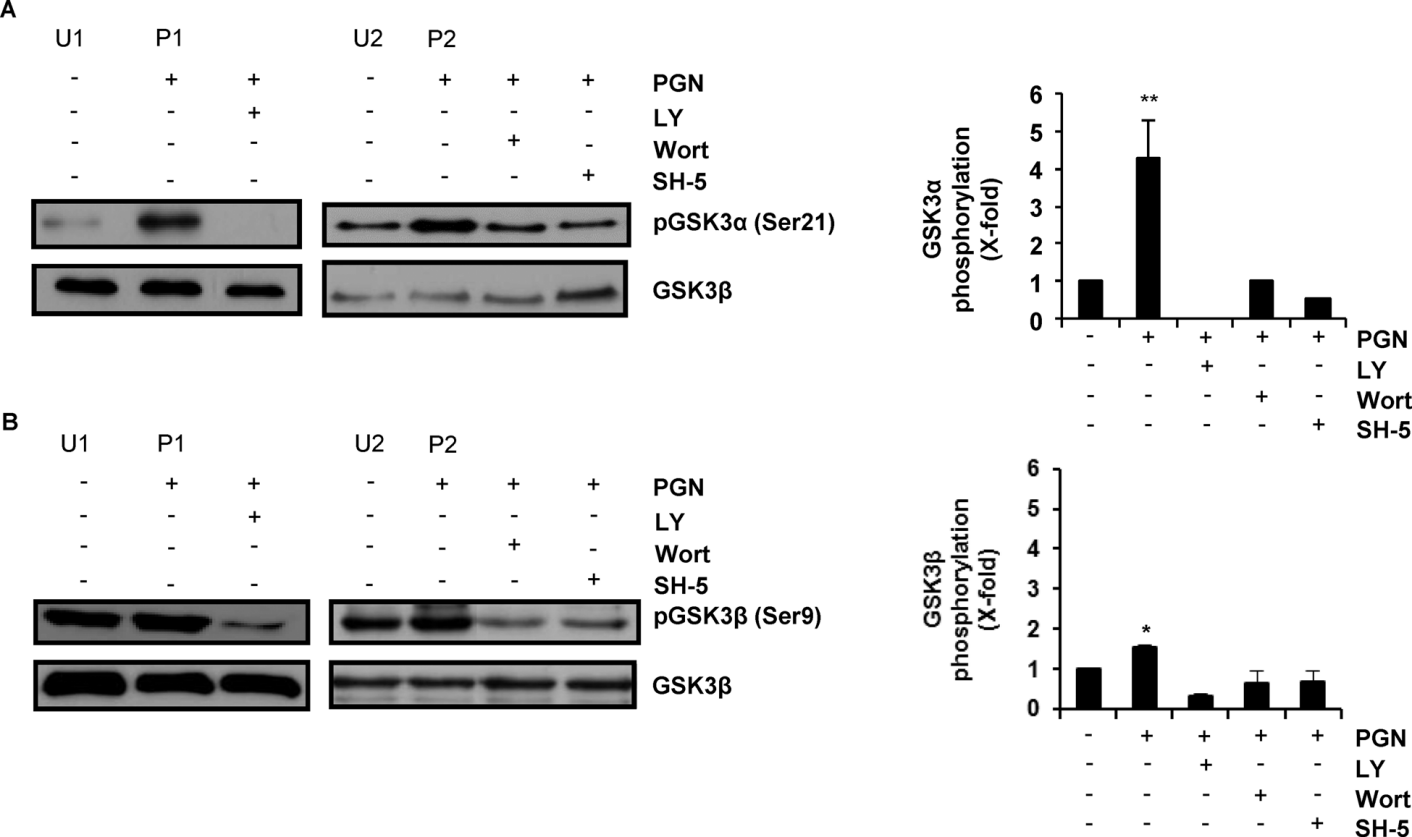
PGN induces PI3K/Akt-dependent phosphorylation of GSK3α and GSK3β in BEC. A-B) BEC were untreated and unstimulated (U1 and U2), stimulated with 10 μg/mL of PGN for 30 min (P1 and P2) or treated with either 10 μM of LY294002 (LY), 100 nM of Wortmannin (Wort) or 10 μM of SH-5 for 30 min, and then stimulated with 10 μg/mL of PGN for 30 min. Protein extracts were analyzed by western blot and probed with monoclonal antibodies against the phosphorylated forms of GSK3α (pGSK3α Ser21) or GSK3β (pGSK3β Ser9. To verify for equal amount of proteins, blots were stripped and reprobed with an antibody that recognizes the nonphosphorylated form of GSK3β. Blots are representative of three independent experiments. Graphs indicate the band intensity obtained by densitometric analysis. In each graph, the densitometric control values plotted were the average of U1 + U2 while the values plotted for the PGN-stimulated cells were the average of P1 + P2. Results are expressed as the mean ± S.E.M. (*n* = 3). *p < 0.05; **p < 0.01, compared with the unstimulated control.

Next, we tested if phosphorylation of GSK3α and GSK3β in endothelial cells stimulated with PGN required activation of PI3K and Akt. Pre-treatment of BEC with LY or Wortmannin (Wort, an inhibitor of PI3K) and SH-5 (an inhibitor of Akt) and stimulated with PGN induced a significant decrease in GSK3α and GSK3β phosphorylation (Fig 2A and 2B). No significant changes in phosphorylation levels were observed when cells were treated with the inhibitors for PI3K (LY), Akt (Akt-i IV) and GSK3 (SB-216763, SB) alone (S3A–S3C Fig). These data indicate that phospho-inhibition of GSK3α and GSK3β in BEC depends on the PI3K and Akt activity.

### Phosphorylation of GSK3α and GSK3β by Akt in BEC Stimulated with PGN Inhibited the Ability of both Isoforms to Phosphorylate Glycogen Synthase

**Fig 3:**
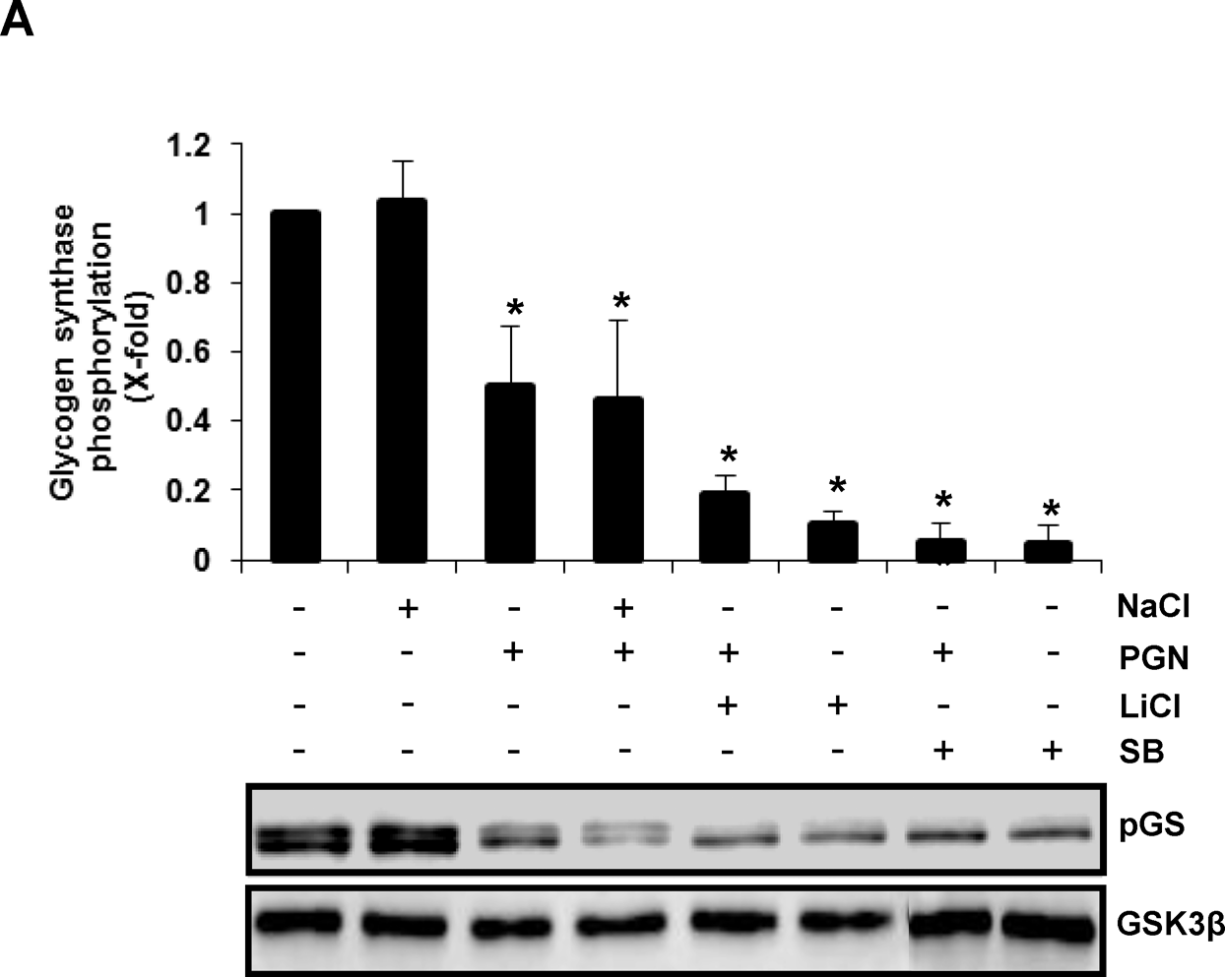
Inhibition of glycogen synthase phosphorylation at Ser641 in BEC treated with PGN, LiCl or SB, and stabilization of β-catenin levels by GSK3α or GSK3β gene silencing. A) BEC were either stimulated with 10 μg/mL of PGN for 30 min, treated with 10 mM NaCl for 60 min, 10 mM LiCl or 10 μM SB and then stimulated with 10 μg/mL of PGN for 30 min. As controls BEC were left untreated (-) or incubated with NaCl, LiCl or SB alone for 60 min. B) BEC were transfected with siRNA targeting GSK3α (siRNA GSK3α), siRNA targeting GSK3β (siRNA GSK3β), control siRNA (siRNA control) or left untransfected (-) for 16 days. After transfection, BEC were incubated for 24 h. Protein extracts were analyzed by western blot and probed with a monoclonal antibody against the phosphorylated form of glycogen synthase (GS Ser641) or the nonphosphorylated form of β-catenin. To verify that equal amount of protein was loaded in each lane, blots were stripped and reprobed with antibodies that recognize the nonphosphorylated forms of GSK3β (A) or β-actin (B). Blots are representative of three independent experiments. Graphs indicate the band intensity obtained by densitometric analysis. Results are expressed as the mean ± S.E.M. (*n* = 3). *p<0.05, compared with the untreated (A) or untransfected (B) control.

To test if phosphorylation of GSK3α at Ser21 and GSK3β at Ser9 resulted in the inhibition of their enzymatic activity, we evaluated the phosphorylation of glycogen synthase (GS) at Ser641, one of the downstream targets of both isoforms. Incubation of BEC with PGN caused the inhibition of 40% to 60% GS phosphorylation, compared with the untreated control (Fig 3). Pre-treatment of BEC with LiCl or SB (two inhibitors of GSK3), SB plus PGN or LiCl plus PGN caused an even stronger reduction in GS phosphorylation. These effects were not due to an osmolarity effect because treatment of BEC with 10 mM NaCl did not change the phosphorylation level of GS in cells not stimulated or stimulated with PGN (Fig 3). These data indicate that phosphorylation of GSK3 in BEC stimulated with PGN induced a significant reduction of GSK3 activity.

### PGN Regulates the Expression of IL-12p40 through a Mechanism that Involves TLR2/Akt Activation and GSK3 Inhibition

**Fig 4:**
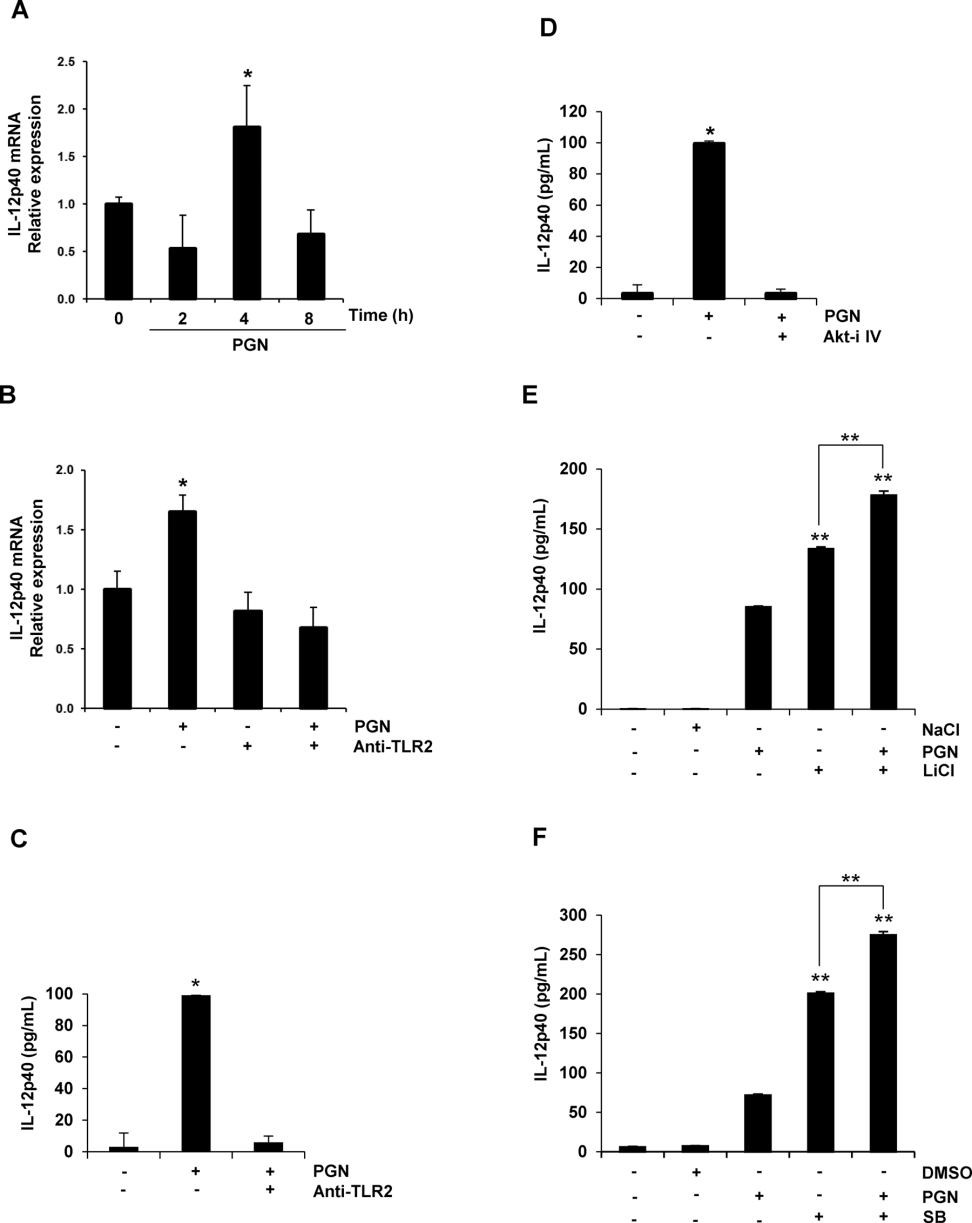
PGN induces IL-12p40 expression through TLR2/Akt activation and GSK3 inhibition in BEC. A) BEC were left untreated and unstimulated (0) or stimulated with 10 μg/mL of PGN-prep for 2, 4 or 8 h. B) BEC were stimulated with 10 μg/mL of PGN for 4 h, treated with 5 μg/mL of neutralizing anti-TLR2 for 60 min, or treated with 5 μg/mL of anti-TLR2 for 60 min and then stimulated with 10 μg/mL of PGN for 4 h. C) BEC were stimulated with 10 μg/mL of PGN for 9 h or treated with 5 μg/mL of anti-TLR2 for 60 min and then stimulated with 10 μg/mL of PGN for 9 h. D) BEC were stimulated with 10 μg/mL of PGN for 9 h or pretreated with 1 μM of Akt inhibitor IV (Akt-i IV) for 30 min and then stimulated with 10 μg/mL for 9 h. E) BEC were treated with 10 mM NaCl for 60 min, stimulated with 10 μg/mL of PGN for 9 h, treated with 10 mM of LiCl for 60 min or treated with 10 mM of LiCl for 60 min and then stimulated with 10 μg/mL of PGN for 9 h. F) BEC were treated with 10 μM of DMSO, stimulated with 10 μg/mL of PGN for 9 h, treated with 10 μM of SB 216763 (SB) for 30 min or treated with 10 μM of SB 216763 (SB) for 30 min and then stimulated with 10 μg/mL of PGN for 9 h. As controls, in B-F BEC were left untreated and stimulated (-). Total RNA was extracted and relative transcript level of IL-12p40 was quantitated by qRT-PCR using the delta-delta Ct method, and amplification of β-actin as a reference gene (A-B). Cell-free supernatants were analyzed by ELISA for production of IL-12p40 (C-F). Results are expressed as the mean ± S.E.M. (*n* = 3). *p <0.05; **p <0.01. All data were compared with the untreated and unstimulated controls.

One of the most important cytokines produced during the initial stages of the inflammatory response is the IL-12p40. The expression of this cytokine in macrophages stimulated with different PAMPs is down-regulated due to the inhibitory effect on GSK3β activity [32]. Therefore, we determined if IL-12p40 expression is regulated in a similar manner in endothelial cells stimulated with PGN from *S*. *aureus*. When transcript levels of IL-12p40 were analyzed in BEC stimulated with PGN we observed an increase in IL-12p40 mRNA at 4 h, which decreased to the control level at 8 h (Fig 4A). This increase was dependent on TLR2 activation because treatment of BEC with a neutralizing anti-TLR2 reduced the IL-12p40 to the level of control mRNA (Fig 4B and 4C). Inhibition of Akt activity strongly reduced the IL-12p40 protein levels (Fig 4D). In contrast, inhibition of GSK3 activity with LiCl or SB caused a strong increase in IL-12p40 protein amounts (Fig 4E and 4F). These results indicate that IL-12p40 expression in BEC stimulated with PGN was associated with activation of TLR2/Akt and inhibition of GSK3.

GSK3 can both positively and negatively affect different transcription factors such as NF-κB and CREB that are responsible for the regulation of pro- and anti- inflammatory cytokine production, respectively [32]. Thus, we tested the relative abundance of NF-κB p65 subunit phosphorylation at Ser536 (one of the phosphorylation sites at the transactivation domain) when both isoforms of GSK3 were inhibited with LiCl and stimulated with PGN. Data obtained show a ∼5-fold increase in p65 phosphorylation in BEC stimulated with PGN alone and an even stronger increase (∼9 fold) in BEC pre-treated with LiCl (S4A Fig). Pretreatment of BEC with NaCl was included to rule out any osmolarity effect (S4A Fig). Next, we performed GSK3α/β gene silencing. Surprisingly, siRNA gene silencing of either GSK3α or GSK3β in BEC stimulated with PGN produced a strong increase in p65 phosphorylation (S4B Fig). We reproducibly observed less phospho-p65 relative abundance when expression of GSK3β was silenced by siRNA. Perhaps, this may imply that phosphorylation at Ser536 contributes differentially not only to the expression of IL-12p40 but also to other NF-κB-regulated genes.

### Inhibition of GSK3α Activity is Responsible for IL-12p40 Increase

**Fig 5:**
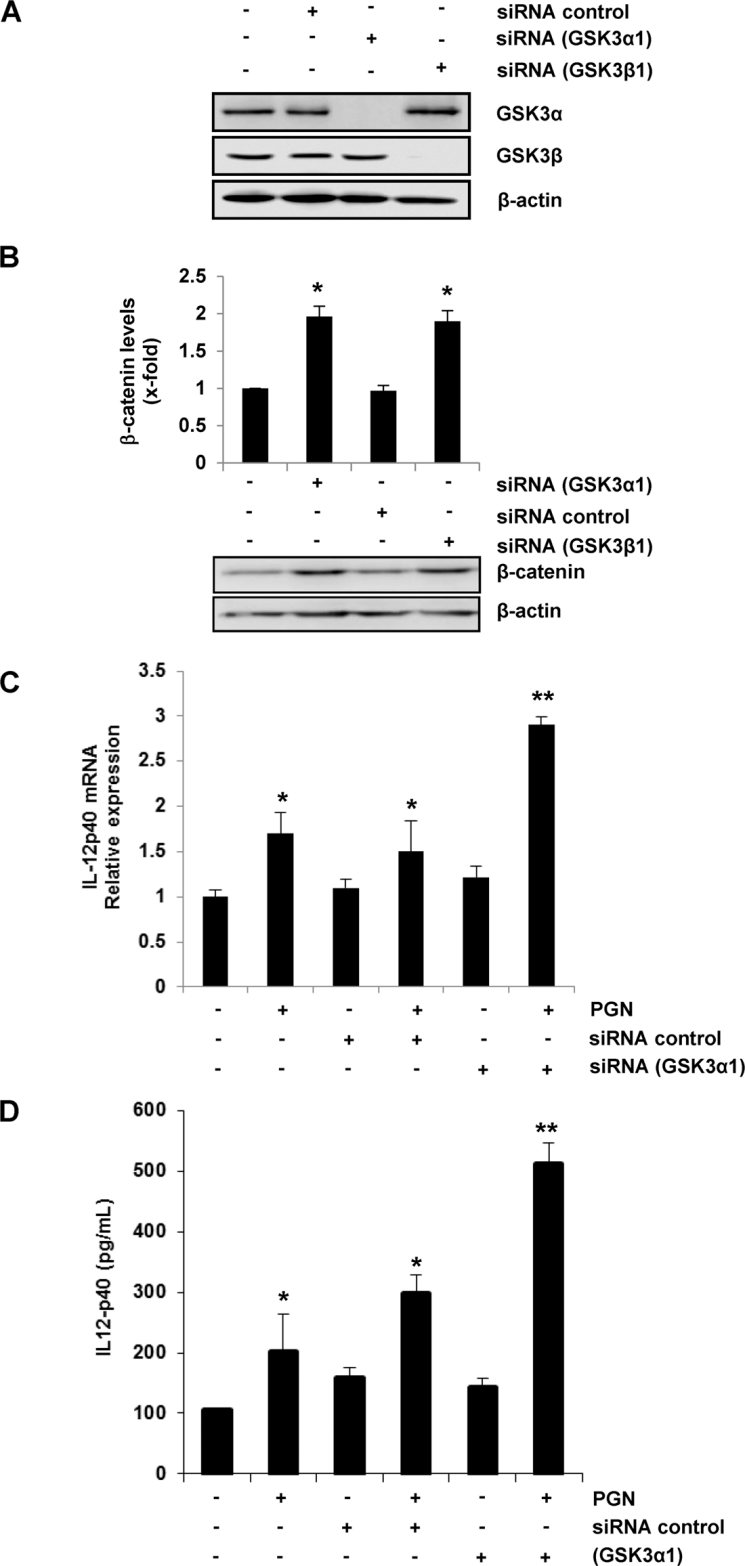
GSK3α inhibition up-regulates IL-12p40 expression in BEC stimulated with PGN. A) BEC were left untransfected (-), transfected with control siRNA (siRNA control), transfected with siRNA targeting GSK3α (siRNA GSK3α1) or transfected with siRNA targeting GSK3β (siRNA GSK3β1). B) BEC were left untransfected and unstimulated (-), stimulated with 10 μg/mL of PGN for 4 h, transfected with control siRNA (siRNA control), transfected with control siRNA and then stimulated with 10 μg/mL PGN for 4 h, transfected with siRNA targeting GSK3α (siRNA GSK3α1) or transfected with siRNA targeting GSK3α (siRNA GSK3α1) and then stimulated with 10 μg/mL of PGN for 4 h. C) BEC were left untransfected and unstimulated (-), stimulated with 10 μg/mL of PGN for 9 h, transfected with control siRNA (siRNA control), transfected with control siRNA and then stimulated with 10 μg/mL of PGN for 9 h, transfected with siRNA targeting GSK3α (siRNA GSK3α1) or transfected with siRNA targeting GSK3α1 and then stimulated with 10 μg/mL of PGN for 9 h. Protein extracts were analyzed by western blot and probed with a monoclonal antibody against the phosphorylated forms of GSK3α and GSK3β (A). To verify that equal amount of proteins was loaded in each lane, blots were stripped and reprobed with an antibody that recognizes the nonphosphorylated form of β-actin (A). Blots are representative of three independent experiments. Total RNA was extracted and relative transcript level of IL-12p40 was quantitated by qRT-PCR, using the delta-delta Ct method and amplification of β-actin as a reference gene (B). Cell-free supernatants were analyzed by ELISA for production of IL-12p40 (C). Results are expressed as the mean ± S.E.M. (*n* = 3). *p <0.05; **p <0.01. All data were compared with the untreated and untransfected controls.

A number of reports have confirmed that GSK3β is the isoform involved in the regulation of pro- and anti-inflammatory cytokines expression. Such experimental evidence, along with the fact that in our case we consistently observed that GSK3α was the isoform more phosphorylated when BEC were stimulated with PGN, prompted us to explore which GSK3 isoform was responsible for the IL-12p40 increase. First, we obtained the evidence of gene silencing for each isoform (Fig 5A). Next, as another control of GSK3α/β gene silencing, we measured the total (cytoplasmic and nuclear) relative abundance of β–catenin because it is known that in resting cells the constitutive activity of GSK3 negatively regulates the Wnt/β-catenin signaling. That is GSK3-dependent phosphorylation of β-catenin promotes its ubiquitylation and subsequent degradation by the proteasome 26S [20]. Thus, when the activity of GSK3 isoforms is inhibited by chemical compounds or the genes of the isoforms are silenced by siRNA treatment, the phosphorylation of β-catenin is blocked and this in turn induces an intracellular accumulation of β-catenin. Transfection of BEC with siRNA targeting GSK3α (siRNA GSK3α) or with siRNA targeting GSK3β (siRNA GSK3β) affected the activity of GSK3 because we detected an increase in total β-catenin levels compared with un-transfected or transfected BEC with control siRNA (siRNA control) (Fig 5B, S1 Dataset). These data demonstrate that the activity GSK3α and GSK3β regulates the stability of β–catenin in BEC, as it has been confirmed in other types of cells [20].

**Fig 6:**
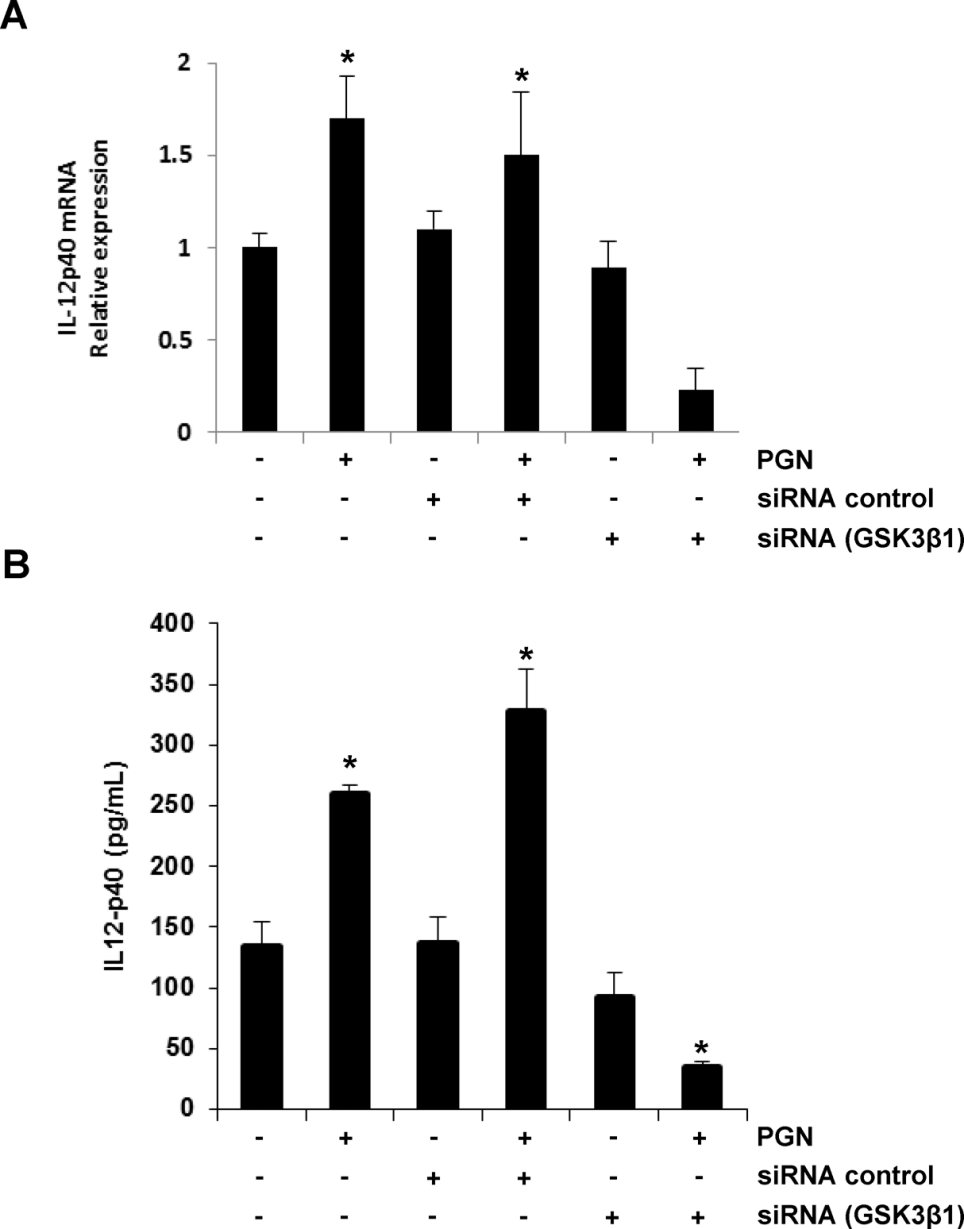
GSK3β inhibition down-regulates IL-12p40 expression in BEC stimulated with PGN. A) BEC were left untransfected and unstimulated (-), stimulated with 10 μg/mL of PGN for 4 h, transfected with control siRNA (siRNA control), transfected with control siRNA and then stimulated with 10 μg/mL of PGN for 4 h, transfected with siRNA targeting GSK3β (siRNA GSK3β1) or transfected with siRNA targeting GSK3β (siRNA GSK3β1) and then stimulated with 10 μg/mL of PGN for 4 h. B) BEC were left untransfected and unstimulated (-), stimulated with 10 μg/mL of PGN for 9 h, transfected with control siRNA (siRNA control), transfected with control siRNA and then stimulated with 10 μg/mL of PGN for 9 h, transfected with siRNA targeting GSK3β (siRNA GSK3β1) or transfected with siRNA targeting GSK3β (siRNA GSK3β1) and then stimulated with 10 μg/mL of PGN for 9 h. Total RNA was extracted and relative transcript level of IL-12p40 was quantitated by qRT-PCR, using the delta-delta Ct method, and amplification of β-actin as a reference gene (A). Cell-free supernatants were analyzed by ELISA for production of IL-12p40 (B). Results are expressed as the mean ± S.E.M. (*n* = 3). *p <0.05. All data were compared with the untreated and untransfected controls.

Analysis of IL-12p40 expression at the level of mRNA and protein showed a significant increase in BEC transfected with siRNA GSK3α and stimulated with PGN, compared with BEC stimulated with PGN alone or BEC transfected with siRNA control and stimulated with PGN (Fig 5C and 5D, S2 Dataset). On the other hand, and according to Martin et al. (2005) [32], a significant down-regulation of IL-12p40 was obtained in BEC transfected with siRNA GSK3β and stimulated with PGN (Fig 6A and 6B, S3 Dataset). To rule out any off-target effect of the siRNA sequence used, we tested other siRNA sequences for each GSK3 isoform. Quantitation of the IL-12p40 protein levels after silencing GSK3α or GSK3β with different siRNA sequences gave comparable values to those previously obtained (S5 Fig, and S4 Dataset). Altogether these results indicate that both GSK3α and GSK3β differentially modulate the expression of IL-12p40 in endothelial cells stimulated with PGN from *S*. *aureus*.

## Discussion

**Fig 7:**
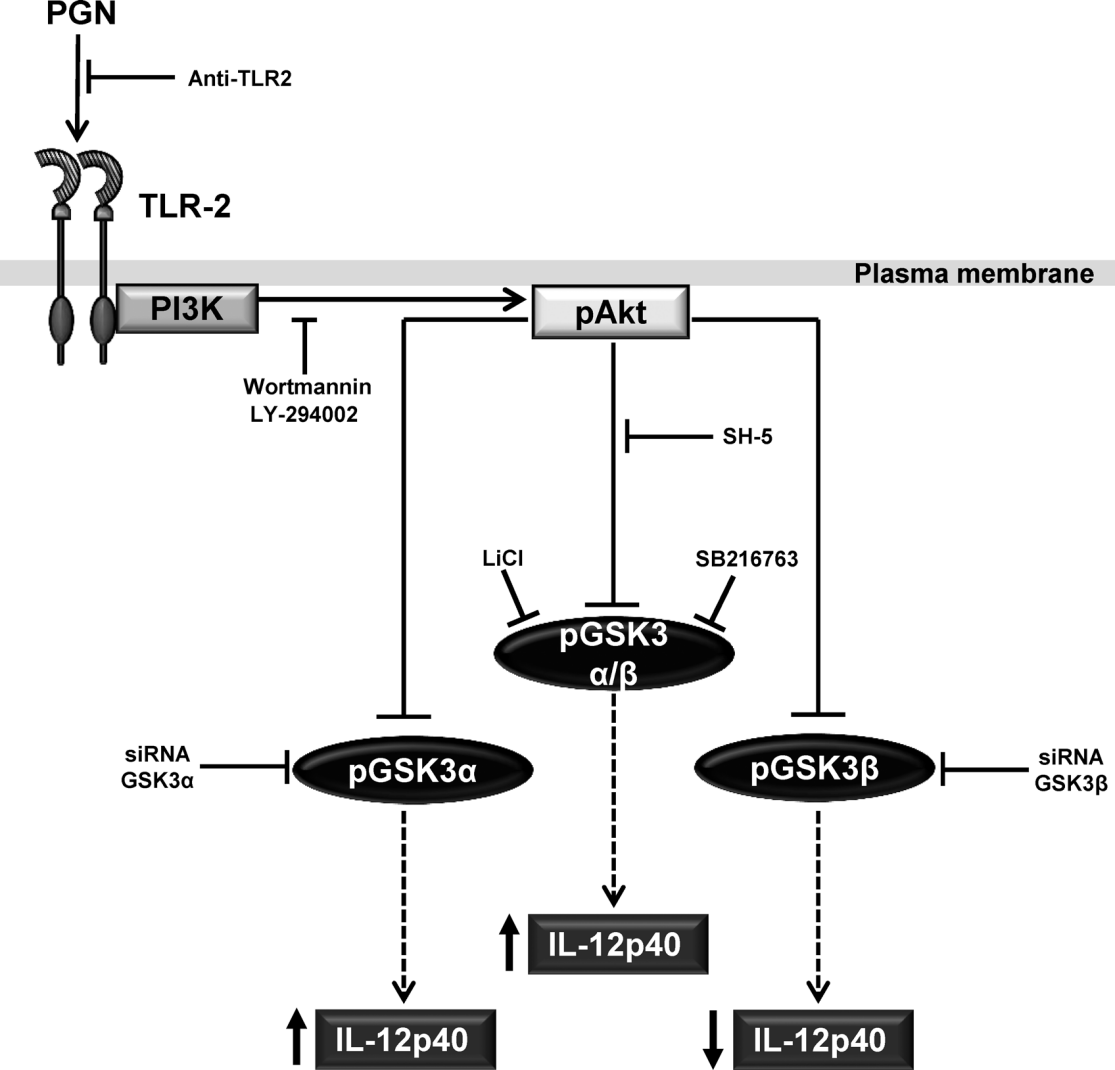
A working model of the signaling pathway involved on the IL-12p40 expression induced by PGN in BEC. PGN induces the activation of the TLR2/PI3K/Akt pathway, which in turn induces GSK3α and GSK3β phosphorylation/inhibition and this in turn up- or down-regulates IL-12p40 expression. Anti-TLR2, Wortmannin (PI3K inhibitor), LY-294002 (PI3K inhibitor) or SH-5 (Akt inhibitor) blocks the phosphorylation/inhibition of GSK3α and GSK3β. LiCl (GSK3 inhibitor), SB-216763 (GSK3 inhibitor) or siRNA GSK3α increases the expression of IL-12p40 while siRNA GSK3β decreases the expression of IL-12p40. Not represented in the diagram is the decrease in IL-12p40 expression when TLR2 is blocked with anti-TLR2 and when Akt is inhibited with Akt-i IV.

The novel findings of this work can be summarized as follows (Fig 7): 1) GSK3 functions as a modulator of the inflammatory response in endothelial cells stimulated with PGN. Previous reports have documented that PGN activates TLR2/PI3K/Akt and induces the expression of pro-inflammatory molecules [3–5]; however, they did not explore how this activation and production of pro-inflammatory cytokines was correlated with changes in GSK3 activity; 2) although it has been accepted that GSK3β is the only isoform that mediates inflammation in cells stimulated with bacterial virulence factors and other stimuli [13] we have found that both GSK3α and GSK3β regulate IL-12p40 expression in endothelial cells stimulated with PGN; 3) the effect of the isoforms on IL-12p40 was different because inhibition of GSK3α activity resulted in the increased expression of IL-12p40 while inhibition of GSK3β activity lead to a decreased expression of the same cytokine and 4) the inhibition of GSK3α and GSK3β was linked to the activation of the TLR2/PI3K/Akt signaling pathway.

Endothelial cell activation during an inflammatory process may be divided into rapid and slow responses that are independent and dependent on new gene expression, respectively [37]. TLRs of endothelial cells play a fundamental role in the regulation of the inflammatory response upon exposure to any of the currently known TLR ligands [38]. PGN, one of the major cell-wall structures of *S*. *aureus*, is an important inducer of the inflammatory response [39]. Although, it is known that activation of the TLR2/PI3K/Akt signaling pathway by PGN induces the activation of NF-κB and the expression of pro-inflammatory molecules such as cytokines, COX2 and iNOS (3, 4), no report has shown that GSK3α or GSK3β inhibition by this signaling pathway regulates pro-inflammatory cytokine expression in response to PGN from *S*. *aureus*. Our findings, using BEC as a model cell, are the first to show that phospho-inhibition of both GSK3α and GSK3β, but predominantly GSK3α, links the TLR2/PI3K/Akt signaling pathway activation with phosphorylation of the NF-κB p65 subunit at Ser536. Furthermore, the inhibition of both isoforms is associated with a differential regulation of IL-12p40 expression (Figs 4–6), a multifunctional cytokine with important tasks in the innate and adaptive immune responses [26, 27].

A number of reports have documented that TLR2 plays a crucial role in the host response against *S*. *aureus* because knockout mice deficient in TLR2 are highly susceptible to staphylococcal infections [40]. However, the specificity of TLR2 for PGN is still an issue of debate. According to Travassos et al. (2004) [41] highly purified PGN did not activate TLR signaling. In contrast, several other authors have proposed that PGN activates TLR2 [3–6, 42] and studies with PGN from *S*. *aureus* lacking lipidated prelipoproteins have co-localized them with Nod2, TLR2 and TLR4 in keratinocytes from murine oral epithelium and HEK293/hTLR2 cells, demonstrating that staphylococcal PGN, and not the associated lipoproteins, is able to trigger a TLR2 specific immune response [43]. Our data support the notion that PGN activates signaling that are associated with TLR2 activation in BEC because blocking of TLR2 with a TLR2 specific neutralizing antibody inhibited the phosphorylation of Akt, phospho-inhibition of GSK3α and GSK3β (Fig 1) and expression of IL-12p40 (Fig 4). Moreover, the PGN used in this study to stimulate BEC was purified with hot SDS, which eliminates the lipopeptides [39]. In agreement with our work, Zhang et al. (2012) [44] observed that TLR2 was activated in dendritic cells stimulated with heat-killed *Brucella abortus*, and this was an essential step for IL-12p40 induction through the activation of subpathways that regulate TLR9 signaling. In a different report, Satta et al. (2008) [45] detected an induction of TLR2 expression in human endothelial cells that served to amplify the inflammatory response to lipopeptides. This was not the case in our study because levels of TLR2 protein in BEC were not modified by PGN treatment, which means that an increase in TLR2 abundance is not a requirement for IL-12p40 expression in BEC stimulated with PGN from *S*. *aureus*.

Although it is well established that Akt phosphorylates and inactivates GSK3α and GSK3β, as we have observed in this study, Gulen et al. (2012) [22] showed that GSK3α, but not GSK3β, can reversely phosphorylates and suppresses Akt activation in resting Th17 cells. These authors also demonstrated that activation of Th17 treated with IL-1 leads to an increase of IKKi activity and GSK3α phosphorylation at Ser21, promoting Akt-mTOR activation [22]. Previous evidence from our lab indicated that GSK3α and GSK3β phosphorylation, as a consequence of BEC infected by *S*. *aureus*, may be involved in the internalization process and perhaps the inflammatory response caused by this bacterium [25].

In the last few years experimental evidence on the different functions of GSK3α and GSK3β is accumulating [14]. Our data demonstrate that inhibition of both GSK3α and GSK3β activity exerts an opposed function on IL-12p40 expression and this is in part different from data obtained by Martin et al. (2005) [32]. These authors found that inhibition of GSK3β, but no GSK3α, activity by treatment of macrophages with LPS or synthetic lipid-A as specific ligands of TLR4 or LTA from *S*. *pneumoniae* as a specific ligand of TLR2, reduced the expression of IL-12p40 [32]. In contrast, our data clearly indicate that phospho-inhibition of GSK3α by treatment of BEC with PGN from *S*. *aureus* increased IL-12p40 expression. This suggests that although GSK3β is the isoform generally associated with the inflammatory response to bacterial infections, as reported by several authors [13, 32], GSK3α may also play an important role in this process through the regulation of pro-inflammatory cytokine expression. Evidence that GSK3α is important in an inflammatory process was recently reported by Giambelluca et al. (2014) [24]. They found that in human neutrophils, in which the main isoform is GSK3α, the addition of LiCl resulted in a significant postranscritptional up-regulation of TNF-α secretion [24]. Interestingly, our results point out that inhibition of GSK3α activity resulted in a marked transcriptional up-regulation of IL-12p40. One explanation for these opposite results may be that active GSK3β phosphorylates NF-κB at its transactivation domain allowing the expression of IL-12p40. In a different scenario and when the activity of GSK3β is inhibited by phosphorylation at Ser9 or gene silencing, this isoform is unable to phosphorylate and activates NF-κ, causing the decrease in IL-12p40 levels. It is also likely that the type of cell used, the stimulus applied, and even the type of cytokine evaluated may explain the mechanistic differences between our results and those reported by Martin et al. (2005) [32] and Giambelluca et. al. (2014) [24]

IL-12 is a cytokine required for innate immune defense and adaptive immunity to pathogens because stimulation of peripheral blood lymphocytes and NK cells with IL-12 produced as a result of infection, induces IFN-γ secretion and increases the cytotoxicity activity as well as proliferation of these cells [26, 27]. Interestingly, it has been proposed the existence of an IL-12-regulated circuit between endothelium and lymphocytes through IFN-γ, resulting in a reciprocal modulation of cellular responses [46, 47]. Moreover, it is likely that IL-12p40 produced by endothelium recruits macrophages to the site of infection because this cytokine has been shown to have chemotactic properties [48]. Although IL-12p40 expression by CD154 stimulation was already detected in endothelium [30], we are the first to demonstrate expression of this cytokine in endothelial cells stimulated with a bacterial structure. Other authors have detected expression of IL-12-related molecules, but not expression of IL-12p40 in human intestinal microvascular endothelial cells stimulated with pro-inflammatory compounds (TNF-α, IFN-γ, IL-1β) and microbial structures [LPS, LTA, PGN, CpG-DNA, flagellin, and poly(I:C)] [49]. In this context, our data suggest that production of IL-12p40 by endothelial cells stimulated with PGN might reflect innate and adaptive immune roles of the endothelium in response to Gram positive microbial antigens. More importantly, the fine modulation in the IL-12p40 expression is of paramount importance because this cytokine is critical for host defense; however, excessive increase in its production can cause severe inflammatory disorders [31]. Therefore, it is likely that a switch in the up- and down-regulation of IL-12p40 expression should co-exist, which might depend on the differences in spatio-temporal participation of the two isoforms of GSK3 and the diverse mechanisms controlling their activity such as phophorylation, subcellular distribution and formation of molecular complexes [14, 15]. More experiments will be undoubtedly needed to clarify the mechanistic details of the differential actions of GSK3α and GSK3β on the phosphorylation of NF-κB and the expression of IL-12p40 and other cytokines during the inflammatory response caused by pathogenic bacteria.

## Supporting Information

S1 Fig PGN does not induce changes in the expression of TLR2 but activates PI3K-dependent phosphorylation of Akt in BEC. A) BEC were left untreated and unstimulated (0) or stimulated with 10 μg/mL of PGN for 15, 30, 60, 120 or 240 min. B) BEC were left unstimulated (U) or stimulated with 1, 10, 20 or 30 μg/mL of PGN for 30 min. C) BEC were left unstimulated (U) or stimulated with 10 μg/mL of PGN for 15, 30, 60 or 120 min. D) BEC were left untreated and unstimulated (U), untreated (-) or treated with 10 μM of LY294002 (LY) for 30 min and then stimulated with 10 μg/mL of PGN for 30 min. Protein extracts were analyzed by western blot and probed with a polyclonal antibody against TLR2 (A) or the phosphorylated form of Akt1 (pAkt Ser473) (B-D). To verify that equal amount of proteins was loaded in each lane, blots were stripped and reprobed with antibodies that recognize β-actin (A) or the nonphosphorylated form of Akt (B-D). Blots are representative of three independent experiments. Graphs on the right indicate the band intensity obtained by densitometric analysis. Results are expressed as the mean ± S.E.M. (n = 3). *p <0.05, compared with the unstimulated control.
(PDF)

S2 Fig Temporal course of GSK3α and GSK3β phosphorylation induced by PGN in BEC. A and B) BEC were left unstimulated (0) or stimulated with 10 μg/mL of PGN for 15, 30, 60 or 120 min. Protein extracts were analyzed by western blot and probed with monoclonal antibodies against the phosphorylated forms of GSK3α (pGSK3α Ser21) or GSK3β (pGSK3β Ser9). To verify equal protein loading, blots were stripped and reprobed with an antibody that recognizes the nonphosphorylated form of GSK3β. Blots are representative of three independent experiments. Graphs on the right indicate the band intensity obtained by densitometric analysis. Results are expressed as the mean ± S.E.M. (n = 3). *p <0.05; **p <0.01, compared with the unstimulated control.
(PDF)

S3 Fig Phosphorylation of Akt at Ser473, GSK3α at Ser21 and GSK3β at Ser9 in BEC treated with inhibitors. BEC were left untreated, treated with 10 μM of LY294002 (LY) for 30 min, treated with 1 μM of Akt inhibitor IV (Akt-i IV) for 30 min or treated with 10μM of SB216763 (SB) for 30 min. Untreated cells were incubated with 10 μM of DMSO. Then, total protein from untreated and treated cell was obtained. A) Phosphorylation of Akt at Ser473; B) Phosphorylation of GSK3α at Ser21; C) Phosphorylation of GSK3β at Ser9. Detection of β–actin and GAPDH were used as control of protein loading. Data presented are representative of two independent experiments.
(PDF)

S4 Fig GSK3 inhibition induces phosphorylation of NF-κB. A) BEC were left untreated and unstimulated (-), treated for 60 min with 10 mM of NaCl, stimulated with 10 μg/mL of PGN for 30 min, treated for 60 min with 10 mM of LiCl and then stimulated with 10 μg/mL of PGN for 30 min or treated for 60 min with 10 mM of LiCl. B) BEC were transfected with control siRNA (siRNA control), transfected with siRNA control and then stimulated with 10 μg/mL of PGN for 30 min, transfected with siRNA targeting GSK3α (siRNA GSK3α), transfected with siRNA GSK3α and then stimulated with 10 μg/mL of PGN for 30 min, transfected with siRNA targeting GSK3β (siRNA GSK3β) or transfected with siRNA GSK3β and then stimulated with 10 μg/mL of PGN for 30 min. Protein extracts were analyzed by western blot and probed with monoclonal antibodies against the phosphorylated forms of p65 (NF-κB p65 Ser536). To check for equal amount of proteins, blots were stripped and reprobed with antibodies that recognize the nonphosphorylated forms of p65 (A) or β-actin (B). Blots are representative of three independent experiments. Graphs indicate the band intensity obtained by densitometric analysis. Results are expressed as the mean ± S.E.M. (n = 3). *p < 0.05; **p < 0.01, compared with the unstimulated control.
(PDF)

S5 Fig GSK3α and GSK3β gene silencing differentially regulates IL-12p40 expression in BEC stimulated with PGN. BEC were left untransfected and unstimulated (-), stimulated with 10 μg/mL of PGN for 9 h, transfected with control siRNA (siRNA control), transfected with control siRNA and then stimulated with 10 μg/mL of PGN for 9 h, transfected with siRNA targeting GSK3α (siRNA GSK3α2 or siRNA GSK3α3) or transfected with siRNA targeting GSK3β (siRNA GSK3β1 or siRNA GSK3β2) and then stimulated with 10 μg/mL of PGN for 9 h. A) Cell-free supernatants were analyzed by ELISA for production of IL-12p40 and B) Protein extracts were analyzed by western blot and probed with a monoclonal antibody against the phosphorylated forms of GSK3α and GSK3β. To verify that equal amount of protein was loaded in each lane, blots were stripped and reprobed with an antibody that recognizes the nonphosphorylated form of β-actin. Results are expressed as the mean ± S.E.M. (n = 3). *p <0.05.
(TIF)

S1 Dataset Raw values used to analyze and construct the graph. (XLSX)

S2 Dataset Raw values used to analyze and construct the graph. (XLSX)

S3 Dataset Raw values used to analyze and construct the graph. (XLSX)

S4 Dataset Raw values used to analyze and construct the graph. (XLSX)

## References

[1] Pantosti A. Methicillin-resistant *Staphylococcus aureus* associated with animals and its relevance to human health. Front Microbiol. 2012;3: 127 10.3389/fmicb.2012.00127 22509176 PMC3321498

[2] McDonald C, Inohara N, Núñez G. Peptidoglycan signaling in innate immunity and inflammatory disease. J Biol Chem. 2005;280: 20177–20180. 15802263 10.1074/jbc.R500001200

[3] Chen BC, Kang JC, Lu YT, Hsu MJ, Liao CC, Chiu WT, et al Rac1 regulates peptidoglycan-induced nuclear factor-kappaB activation and cyclooxygenase-2 expression in RAW 264.7 macrophages by activating the phosphatidylinositol 3-kinase/Akt pathway. Mol Immunol. 2009;46: 1179–1188. 10.1016/j.molimm.2008.11.006 19118901

[4] Lin HY, Tang CH, Chen YH, Wei IH, Chen JH, Lai CH, et al Peptidoglycan enhances proinflammatory cytokine expression through the TLR2 receptor, MyD88, phosphatidylinositol 3-kinase/AKT and NF-kappaB pathways in BV-2 microglia. Int Immunopharmacol. 2010;10: 883–891. 10.1016/j.intimp.2010.04.026 20451669

[5] Chiu YC, Lin CY, Chen CP, Huang KC, Tong KM, Tzeng CY, et al Peptidoglycan enhances IL-6 production in human synovial fibroblasts via TLR2 receptor, focal adhesion kinase, Akt, and AP-1- dependent pathway. J Immunol. 2009;183: 2785–2792. 10.4049/jimmunol.0802826 19635908

[6] Chen BC, Chang YS, Kang JC, Hsu MJ, Sheu JR, Chen TL, et al Peptidoglycan induces nuclear factor-κB activation and cyclooxygenase-2 expression via Ras, Raf-1, and ERK in RAW264.7 macrophages. J Biol Chem. 2004;279: 20889–20897. 15007072 10.1074/jbc.M311279200

[7] Liu P, Cheng H, Roberts TM, Zhao JJ. Targeting the phosphoinositide 3-kinase pathway in cancer. Nat Rev Drug Discov. 2009;8: 627–644. 10.1038/nrd2926 19644473 PMC3142564

[8] Wang H, Brown J, Martin M. Glycogen synthase kinase 3: a point of convergence for the host inflammatory response. Cytokine 2011;53: 130–140. 10.1016/j.cyto.2010.10.009 21095632 PMC3021641

[9] Alessi D, Deak M, Casamayor A, Caudwell FB, Morrice N, Norman DG, et al Phosphoinositide-dependent protein kinase-1 (PDK1): structural and functional homology with the *Drosophila* DSTPK61 kinase. Curr Biol. 1997;7: 776–789. 9368760 10.1016/s0960-9822(06)00336-8

[10] Frodin M, Antal TL, Dummler BA, Jensen CJ, Deak M, Gammeltoft S, et al A phosphoserine/threonine-binding pocket in AGC kinases and PDK1 mediates activation by hydrophobic motif phosphorylation. EMBO J. 2002;21: 5396–5407. 12374740 10.1093/emboj/cdf551 PMC129083

[11] Sarbassov DD, Guertin DA, Ali SM, Sabatini DM. Phosphorylation and regulation of Akt/PKB by the rictor-mTOR complex. Science 2005;307: 1098–1010. 15718470 10.1126/science.1106148

[12] Hresko RC, Mueckler M. mTOR-RICTOR is the Ser473 kinase for Akt/protein kinase B in 3T3-L1 adipocytes. J Biol Chem. 2005;280: 40406–40416. 16221682 10.1074/jbc.M508361200

[13] Cortés-Vieyra R, Bravo-Patiño A, Valdez-Alarcón JJ, Cajero-Juárez M, Finlay BB, Baizabal-Aguirre VM. Role of glycogen synthase kinase-3 beta in the inflammatory response caused by bacterial pathogens. J Inflamm (Lond) 2012;9: 23. 22691598 10.1186/1476-9255-9-23 PMC3506434

[14] Beurel E, Grieco SF, Jope RS. Glycogen synthase kinase 3 (GSK3): Regulation, actions, and disease. Phrmacol Therapeut. (2014) 10.1016/j.pharmthera.2014.11.016 PMC4340754 25435019

[15] Jope RS, Johnson GV. The glamour and gloom of glycogen synthase kinase-3. Trends Biochem Sci. 2004;29: 95–102. 15102436 10.1016/j.tibs.2003.12.004

[16] Hoeflich KP, Luo J, Rubie EA, Tsao MS, Jin O, Woodgett JR. Requirement for glycogen synthase kinase-3beta in cell survival and NF-kappaB activation. Nature 2000;406: 86–90. 10894547 10.1038/35017574

[17] Jacobs KM, Bhave SR, Ferraro DJ, Jaboin JJ, Hallahan DE, Thotala D. GSK-3β: A bifunctional role in cell death pathways. Int J Cell Biol. 2012 doi: 10.1155/2012/ 930710 10.1155/2012/930710 PMC3364548 22675363

[18] MacAulay K, Doble BW, Patel S, Hansotia T, Sinclair EM, Drucker DJ, et al Glycogen synthase kinase 3α-specific regulation of murine hepatic glycogen metabolism. Cell Metab. 2007;6: 329–337. 17908561 10.1016/j.cmet.2007.08.013

[19] Patel S, Doble BW, MacAulay K, Sinclair EM, Drucker DJ, Woodgett JR. Tissue-specific role of glycogen synthase kinase 3beta in glucose homeostasis and insulin action. Mol Cell Biol. 2008;28: 6314–6328. 10.1128/MCB.00763-08 18694957 PMC2577415

[20] McManus EJ, Sakamoto K, Armit LJ, Ronaldson L, Shpiro N, Marquez R, et al Role that phosphorylation of GSK3 plays in insulin and Wnt signalling defined by knockin analysis. EMBO J. 2005;24: 1571–1583. 15791206 10.1038/sj.emboj.7600633 PMC1142569

[21] Kaidanovich-Beilin O, Lipina TV, Takao K, van Eede M, Hattori S, Laliberté C, et al Abnormalities in brain structure and behavior in GSK-3alpha mutant mice. Mol Brain 2009;2: 35 10.1186/1756-6606-2-35 19925672 PMC2785804

[22] Gulen MF, Bulek K, Xiao H, Yu M, Gao J, Sun L, et al Inactivation of the enzyme GSK3α by the kinases IKKi promotes AKT-mTOR signaling pathway that mediates interleukin-1-induced Th17 cell maintenance. Immunity 2012;37: 800–812. 10.1016/j.immuni.2012.08.019 23142783 PMC3512562

[23] Barrel WB, Szabo-Rogers HL, Liu KJ. Novel reporter alleles of GSK-3α and GSK-3β. PLoS One 2012;7: e 50422. 10.1371/journal.pone.0050422 PMC3503927 23185619

[24] Giambelluca MS, Bertheau-Mailhot G, Laflamme C, Rollet-Labelle E, Servant MJ, Pouliot M. TNF-α expression in neutrophils and its regulation by glycogen synthase kinase-3: a potentiating role for lithium. FASEB J. 2014;28: 3679–3690. 10.1096/fj.14-251900 24803542

[25] Oviedo-Boyso J, Cortés-Vieyra R, Huante-Mendoza A, Yu HB, Valdez-Alarcón JJ, Bravo-Patiño A, et al The phosphoinositide-3-kinase-Akt signaling pathway is important for *Staphylococcus aureus* internalization by endothelial cells. Infect Immun. 2011;79: 4569–4577. 10.1128/IAI.05303-11 21844240 PMC3257907

[26] Trinchieri G. Interleukin-12: “A proinflammatory cytokine with immunoregulatory functions that bridge innate resistance and antigen-specific adaptive immunity”. Annu Rev Immunol. 1995;13: 251–276. 7612223 10.1146/annurev.iy.13.040195.001343

[27] Watford WT, Moriguchi M, Morinobu A, O'Shea JJ. The biology of IL-12: coordinating innate and adaptive immune responses. Cytokine Growth Factor Rev. 2003;14: 361–368. 12948519 10.1016/s1359-6101(03)00043-1

[28] Kobayashi M, Fitz L, Ryan M, Hewick RM, Clark SC, Chan S, et al Identification and purification of natural killer cell stimulatory factor (NKSF), a cytokine with multiple biologic effects on human lymphocytes. J Exp Med. 1989;170: 827–845. 2504877 10.1084/jem.170.3.827 PMC2189443

[29] Stern AS, Podlaski FJ, Hulmes JD, Pan YC, Quinn PM, Wolitzky AG, et al Purification to homogeneity and partial characterization of cytotoxic lymphocyte maturation factor from human B-lymphoblastoid cells. Proc Natl Acad Sci U.S.A. 1990;87: 6808–6812. 2204066 10.1073/pnas.87.17.6808 PMC54627

[30] Lienenlüke B, Germann T, Kroczek RA, Hecker M. CD154 stimulation of interleukin-12 synthesis in human endothelial cells. Eur J Immunol. 2000;30: 2864–2870. 11069068 10.1002/1521-4141(200010)30:10<2864::AID-IMMU2864>3.0.CO;2-W

[31] Gee K, Guzzo C, Che Mat NF, Ma W, Kumar A. The IL-12 family of cytokines in infection, inflammation an autoimmune disorders. Inflamm Allergy Drug Targets 2009;8: 40–52. 19275692 10.2174/187152809787582507

[32] Martin M, Rehani K, Jope RS, Michalek SM. Toll-like receptor-mediated cytokine production is differentially regulated by glycogen synthase kinase 3. Nat Immunol. 2005;6: 777–784. 16007092 10.1038/ni1221 PMC1933525

[33] Cajero-Juárez M, Ávila B, Ochoa A, Garrido-Guerrero E, Varela-Echavarría A, Martínez de la Escalera G, et al Immortalization of bovine umbilical vein endothelial cells: a model for the study of vascular endothelium. Eur J Cell Biol. 2002;81: 1–8. 11893074 10.1078/0171-9335-00213

[34] Dziarski R, Gupta D. *Staphylococcus aureus* peptidoglycan is a toll-like receptor 2 activator: a reevaluation. Infect Immun. 2005;73: 5212–5216. 16041042 10.1128/IAI.73.8.5212-5216.2005 PMC1201261

[35] Bradford MM. A rapid and sensitive method for the quantitation of microgram quantities of protein utilizing the principle of protein-dye binding. Anal Biochem 1976;72: 248–254. 942051 10.1016/0003-2697(76)90527-3

[36] Konnai S, Usui T, Ohashi K, Onuma M. The rapid quantitative analysis of bovine cytokine genes by real-time RT-PCR. Vet Microbiol. 2003;94: 283–293. 12829382 10.1016/s0378-1135(03)00119-6

[37] Pober JS, Sessa WC. Envolving functions of endothelial cells in inflammation. Nat Rev Immunol. 2007;7: 803–815. 17893694 10.1038/nri2171

[38] Fitzner N, Clauberg S, Essmann F, Liebmann J, Kolb-Bachofen V. Human skin endothelial cells can express all 10 TLR genes and respond to respective ligands. Clin Vaccine Immunol. 2009;15: 138–146. 10.1128/CVI.00257-07 PMC2223852 17978010

[39] Dziarski R, Ulmer AJ, Gupta D. Interactions of CD14 with components of gram positive bacteria. Chem Immunol. 2000;74: 83–107. 10608083 10.1159/000058761

[40] Pietrocola G, Arcicola CR, Rindi S, Di Poto A, Missineo A, Montanaro L, et al Toll.like receptors (TLRs) in innate immune defense agains *Staplylococcus aureus* . Int J Artif Organs 2011;34: 799–810. 10.5301/ijao.5000030 22094559

[41] Travassos LH, Giardin SE, Philpott DJ, Blanot D, Nahori MA, Werts C, et al Toll-like receptor 2-dependent bacterial sensing does not occur via peptidoglycan recognition. EMBO Rep. 2004;5: 1000–1006. 15359270 10.1038/sj.embor.7400248 PMC1299148

[42] Dziarski R, Gupta D. Peptidoglycan recognition in innate immunity. J Endotoxin Res. 2005;11: 304–310. 16263004 10.1179/096805105X67256

[43] Müller-Anstett MA, Müller P, Albrecht T, Nega M, Wagener J, Gao Q, et al Staphylococcal peptidoglycan co-localizes with Nod2 and TLR2 and activates innate immune response via both receptors in primary murine keratinocytes. PLoS One 2010;5: e13153 10.1371/journal.pone.0013153 20949035 PMC2951902

[44] Zhang CY, Bai N, Zhang ZH, Liang N, Dong L, Xiang R, et al TLR2 signaling subpathways regulate TLR9 signaling for the effective induction of IL-12 upon stimulation by heat-killed *Brucella abortus* . Cell Mol Immunol. 2012;9: 324–333. 10.1038/cmi.2012.11 22635254 PMC4012865

[45] Satta N, Kruithof EK, Reber G, Moerloose P. Induction of TLR2 expression by inflammatory stimuli is required for endothelial cell responses to lipopetides. Mol Immunol. 2008;46: 145–157. 10.1016/j.molimm.2008.07.017 18722665

[46] Strasly M, Cavallo F, Geuna M, Mitola S, Colombo MP, Forni G, et al IL-12 inhibition of endothelial cell functions and angiogenesis depends on lymphocyte-endothelial cell cross-talk. J Immunol. 2001;166: 3890–3899. 11238633 10.4049/jimmunol.166.6.3890

[47] Mitola S, Strasly M, Prato M, Ghia P, Bussolino F. IL-12 regulates an endothelial cell-lymphocyte network: effect on metalloproteinase-9 production. J Immunol. 2003;171: 3725–3733. 14500672 10.4049/jimmunol.171.7.3725

[48] Ha SJ, Lee CH, Lee SB, Kim CM, Jang KL, Shin HS, et al A novel function of IL-12p40 as a chemotactic molecule for macrophages. J Immunol. 1999;163: 2902–2908. 10453037

[49] Heidemann J, Rüther C, Kebschull M, Domschke W, Brüwer M, Koch S, et al Expression of IL-12 related molecules in human intestinal microvascular endothelial cells is regulated by TLR3. Am J Physiol Gastrointest Liver Physiol 2007;293: G1315–G1324. 17947455 10.1152/ajpgi.00142.2007

