# Supplementary figures and images for: The Glycogen Synthase Kinase 3α and β Isoforms Differentially Regulates Interleukin-12p40 Expression in Endothelial Cells Stimulated with Peptidoglycan from Staphylococcus aureus
Source: PLoS One. 2015 Jul 22;10(7):e0132867. doi: 10.1371/journal.pone.0132867 (PMC4511647; doi:10.1371/journal.pone.0132867)

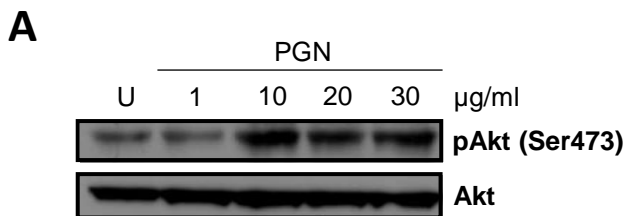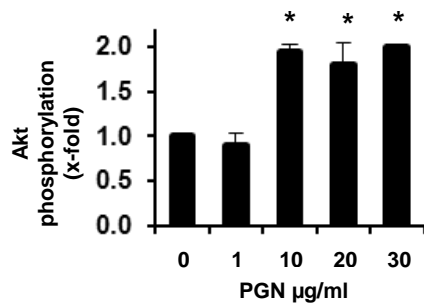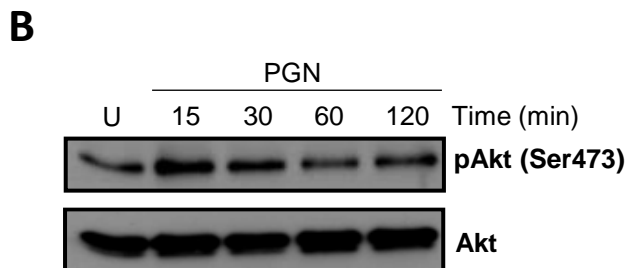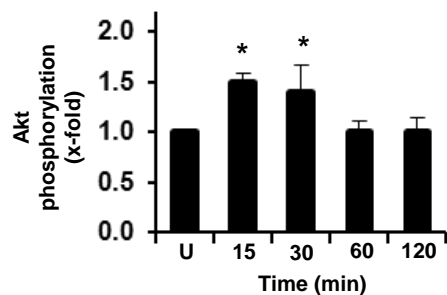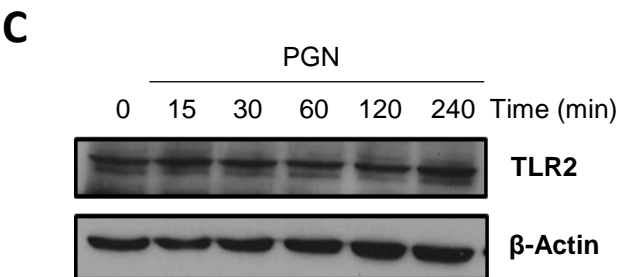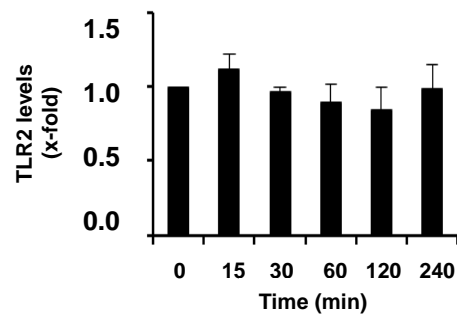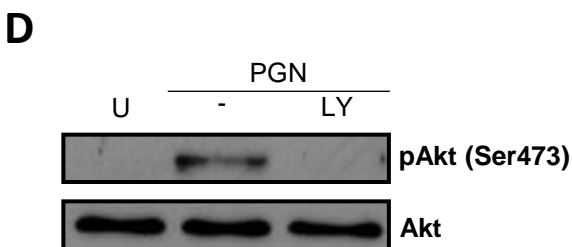

Supplement: S1 Fig — A) BEC were left untreated and unstimulated (0) or stimulated with 10 μg/mL of PGN for 15, 30, 60, 120 or 240 min. B) BEC were left unstimulated (U) or stimulated with 1, 10, 20 or 30 μg/mL of PGN for 30 min. C) BEC were left unstimulated (U) or stimulated with 10 μg/mL of PGN for 15, 30, 60 or 120 min. D) BEC were left untreated and unstimulated (U), untreated (-) or treated with 10 μM of LY294002 (LY) for 30 min and then stimulated with 10 μg/mL of PGN for 30 min. Protein extracts were analyzed by western blot and probed with a polyclonal antibody against TLR2 (A) or the phosphorylated form of Akt1 (pAkt Ser473) (B-D). To verify that equal amount of proteins was loaded in each lane, blots were stripped and reprobed with antibodies that recognize β-actin (A) or the nonphosphorylated form of Akt (B-D). Blots are representative of three independent experiments. Graphs on the right indicate the band intensity obtained by densitometric analysis. Results are expressed as the mean ± S.E.M. (n = 3). *p <0.05, compared with the unstimulated control. (PDF) [file pone.0132867.s001.pdf]

**A**

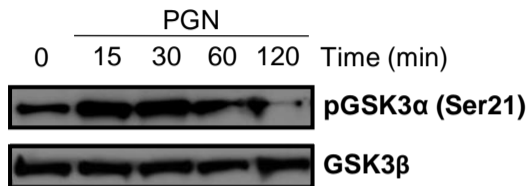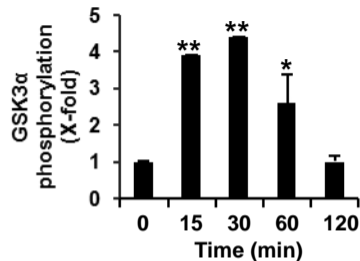

**B**

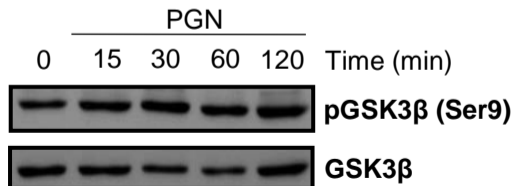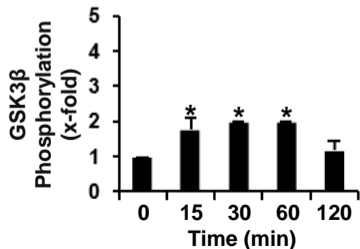

Supplement: S2 Fig — A and B) BEC were left unstimulated (0) or stimulated with 10 μg/mL of PGN for 15, 30, 60 or 120 min. Protein extracts were analyzed by western blot and probed with monoclonal antibodies against the phosphorylated forms of GSK3α (pGSK3α Ser21) or GSK3β (pGSK3β Ser9). To verify equal protein loading, blots were stripped and reprobed with an antibody that recognizes the nonphosphorylated form of GSK3β. Blots are representative of three independent experiments. Graphs on the right indicate the band intensity obtained by densitometric analysis. Results are expressed as the mean ± S.E.M. (n = 3). *p <0.05; **p <0.01, compared with the unstimulated control. (PDF) [file pone.0132867.s002.pdf]

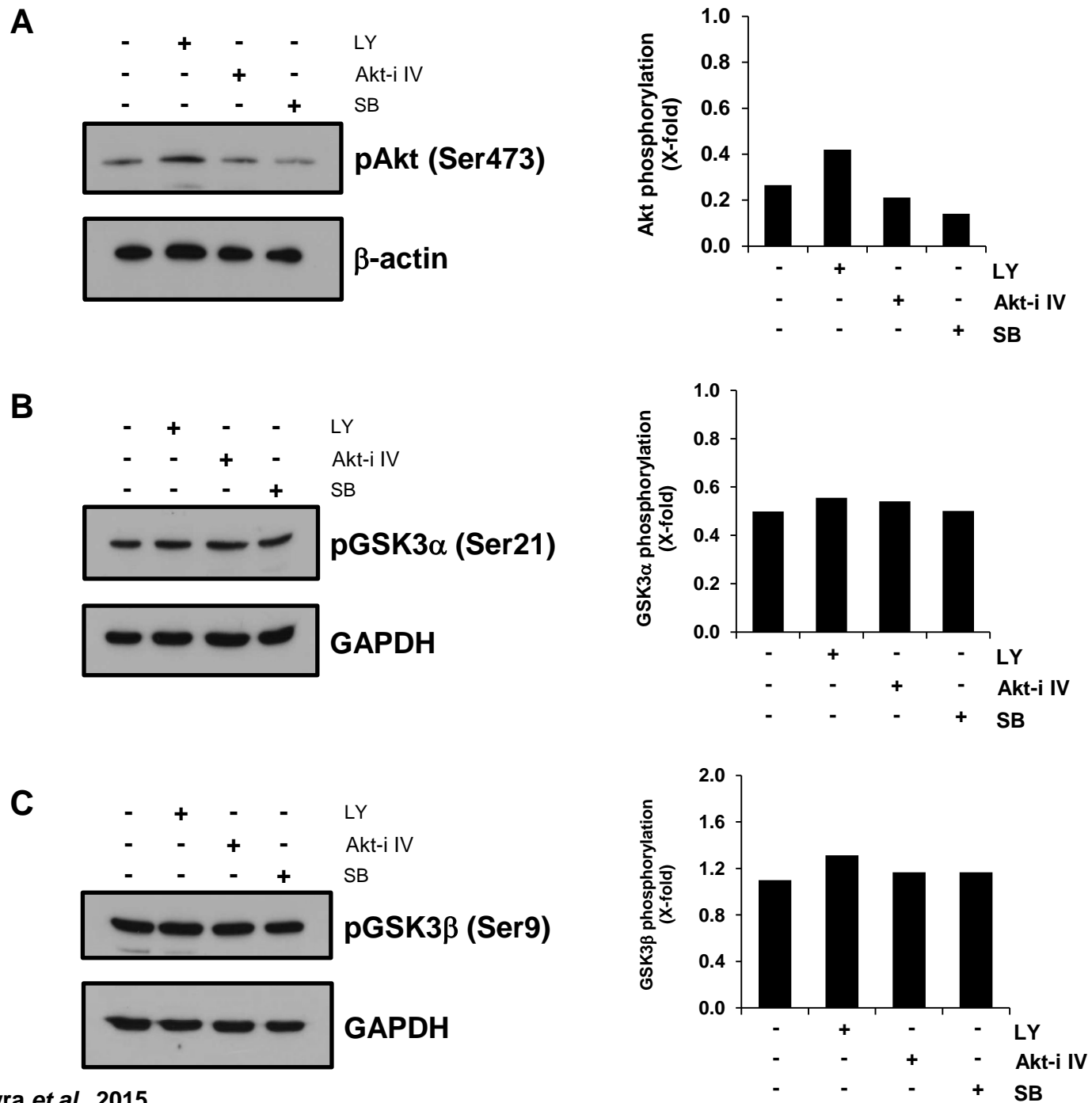

Figure S3. Cortés-Vieyra *et al.*, 2015

Supplement: S3 Fig — BEC were left untreated, treated with 10 μM of LY294002 (LY) for 30 min, treated with 1 μM of Akt inhibitor IV (Akt-i IV) for 30 min or treated with 10μM of SB216763 (SB) for 30 min. Untreated cells were incubated with 10 μM of DMSO. Then, total protein from untreated and treated cell was obtained. A) Phosphorylation of Akt at Ser473; B) Phosphorylation of GSK3α at Ser21; C) Phosphorylation of GSK3β at Ser9. Detection of β–actin and GAPDH were used as control of protein loading. Data presented are representative of two independent experiments. (PDF) [file pone.0132867.s003.pdf]

**A**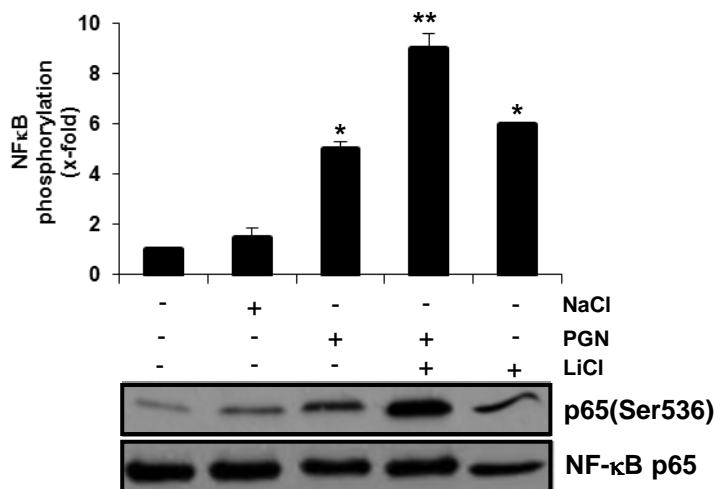**B**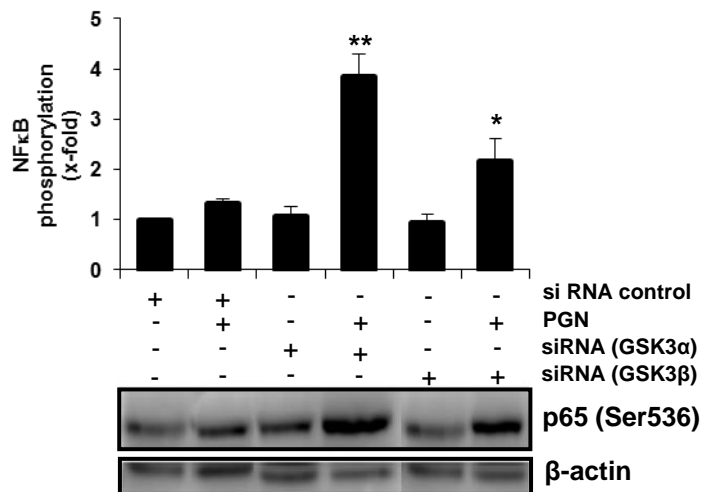

Supplement: S4 Fig — A) BEC were left untreated and unstimulated (-), treated for 60 min with 10 mM of NaCl, stimulated with 10 μg/mL of PGN for 30 min, treated for 60 min with 10 mM of LiCl and then stimulated with 10 μg/mL of PGN for 30 min or treated for 60 min with 10 mM of LiCl. B) BEC were transfected with control siRNA (siRNA control), transfected with siRNA control and then stimulated with 10 μg/mL of PGN for 30 min, transfected with siRNA targeting GSK3α (siRNA GSK3α), transfected with siRNA GSK3α and then stimulated with 10 μg/mL of PGN for 30 min, transfected with siRNA targeting GSK3β (siRNA GSK3β) or transfected with siRNA GSK3β and then stimulated with 10 μg/mL of PGN for 30 min. Protein extracts were analyzed by western blot and probed with monoclonal antibodies against the phosphorylated forms of p65 (NF-κB p65 Ser536). To check for equal amount of proteins, blots were stripped and reprobed with antibodies that recognize the nonphosphorylated forms of p65 (A) or β-actin (B). Blots are representative of three independent experiments. Graphs indicate the band intensity obtained by densitometric analysis. Results are expressed as the mean ± S.E.M. (n = 3). *p < 0.05; **p < 0.01, compared with the unstimulated control. (PDF) [file pone.0132867.s004.pdf]

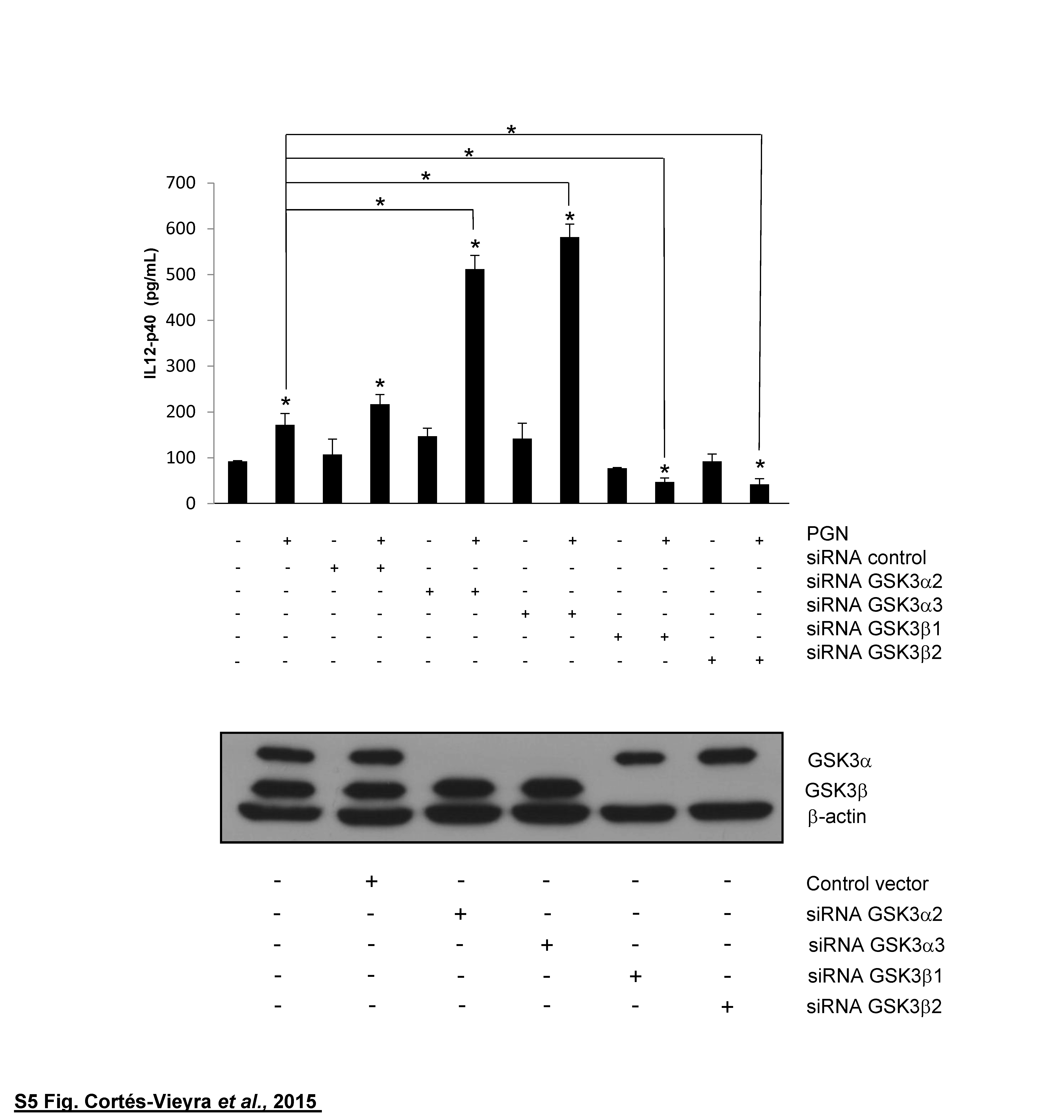

Supplement: S5 Fig — BEC were left untransfected and unstimulated (-), stimulated with 10 μg/mL of PGN for 9 h, transfected with control siRNA (siRNA control), transfected with control siRNA and then stimulated with 10 μg/mL of PGN for 9 h, transfected with siRNA targeting GSK3α (siRNA GSK3α2 or siRNA GSK3α3) or transfected with siRNA targeting GSK3β (siRNA GSK3β1 or siRNA GSK3β2) and then stimulated with 10 μg/mL of PGN for 9 h. A) Cell-free supernatants were analyzed by ELISA for production of IL-12p40 and B) Protein extracts were analyzed by western blot and probed with a monoclonal antibody against the phosphorylated forms of GSK3α and GSK3β. To verify that equal amount of protein was loaded in each lane, blots were stripped and reprobed with an antibody that recognizes the nonphosphorylated form of β-actin. Results are expressed as the mean ± S.E.M. (n = 3). *p <0.05. (TIF) [file pone.0132867.s005.tif]
